# Quantifying Protein–Protein Interactions by Molecular Counting with Mass Photometry

**DOI:** 10.1002/anie.202001578

**Published:** 2020-04-02

**Authors:** Fabian Soltermann, Eric D. B. Foley, Veronica Pagnoni, Martin Galpin, Justin L. P. Benesch, Philipp Kukura, Weston B. Struwe

**Affiliations:** ^1^ Physical and Theoretical Chemistry Department of Chemistry University of Oxford South Parks Road Oxford OX1 3TA UK

**Keywords:** antibodies, mass photometry, protein–protein interactions, receptors, single-molecule studies

## Abstract

Interactions between biomolecules control the processes of life in health and their malfunction in disease, making their characterization and quantification essential. Immobilization‐ and label‐free analytical techniques are desirable because of their simplicity and minimal invasiveness, but they struggle with quantifying tight interactions. Here, we show that mass photometry can accurately count, distinguish by molecular mass, and thereby reveal the relative abundances of different unlabelled biomolecules and their complexes in mixtures at the single‐molecule level. These measurements determine binding affinities over four orders of magnitude at equilibrium for both simple and complex stoichiometries within minutes, as well as the associated kinetics. These results introduce mass photometry as a rapid, simple and label‐free method for studying sub‐micromolar binding affinities, with potential for extension towards a universal approach for characterizing complex biomolecular interactions.

## Graphical abstract

**Keeping count**: Reported is the quantification of protein–protein interactions with light using mass photometry, which detects, images, and quantifies light scattering of single proteins upon binding to an illuminated glass‐water interface. The resulting signals are proportional to the molecular mass of each species and overall abundance, providing a new means to measure the relative abundances of proteins and their complexes, thereby enabling measurement of binding affinities and kinetics by molecular counting.

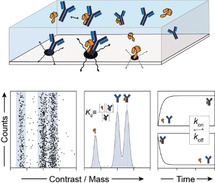

Understanding how biomolecules interact with each other is central to the life sciences. The complexity thereof ranges from specific binary interactions, such as between antibodies and antigens,^1^, ^2^, ^3^ to the formation of complex macromolecular machines.^4^, ^5^ Conversely, undesired interactions are often associated with disease, such as the formation of protein aggregates in neurodegenerative disease,^6^ or the engagement of a virus with its target cell.^7^, ^8^ The high specificity and critical role of these interactions make them an ideal target for intervention, either in promoting a certain response by presenting an alternative binding partner, or preventing (dis)assembly.^9^, ^10^, ^11^ This diversity comes with a broad range of binding strengths and dynamics, measured in terms of thermodynamic and kinetic quantities such as equilibrium constants (e.g. for dissociation, *K*_d_), free energies, and rate constants (*k*_off_ and *k*_on_).

In broad terms, existing biophysical methods can be categorized into size‐based approaches performing quantification and separation by either size or diffusion coefficient, physical interaction with functionalized surfaces, direct mass measurement, enthalpy changes, or light scattering.^12^, ^13^, ^14^, ^15^, ^16^, ^17^ These ensemble‐based methods are complemented by fluorescence‐based approaches^18^ capable of operating at the single‐molecule level, providing additional information on sample heterogeneity and dynamics.^19^, ^20^ All of the above methods operate in the context of various practical shortcomings such as non‐native environments, artefacts caused by protein immobilization and labelling, lack of sensitivity at low concentrations, or lack of resolution.^21^, ^22^, ^23^ Biological systems can pose additional challenges from either particularly fast or slow kinetics to complexities arising from multiple co‐existing species. Label‐free methods struggle with strong binding affinities (*K*_d_<μm), which are often encountered for interactions of relevance for biopharmaceuticals in the context of antibody‐based drugs.^24^

We have recently developed mass photometry (MP), originally introduced as interferometric scattering mass spectrometry (iSCAMS), as a means for detecting and measuring the mass of single proteins and the complexes they form in solution.^26^ MP detects single biomolecules by their light scattering as they bind nonspecifically to a microscope cover glass surface. Each binding event leads to a change in refractive index at the glass/water interface, which effectively alters the local reflectivity and can be detected with high accuracy by taking advantage of optimized interference between scattered and reflected light (Figure 1 a).^25^ The reflectivity change is proportional to the molecular mass, with up to 20 kDa mass resolution and 2 % mass accuracy by calibration with biomolecules of known mass.^26^ Both the original^26^ and subsequent studies have proposed methods to extract binding affinities from MP distributions of biomolecular mixtures,^27^ and shown that the results agree broadly with alternative approaches.^26^, ^27^, ^28^ The degree to which these MP distributions are indeed quantitative, and how they can be used to efficiently extract not only binding affinities but also kinetics, however, remain unexplored.

**Figure 1:**
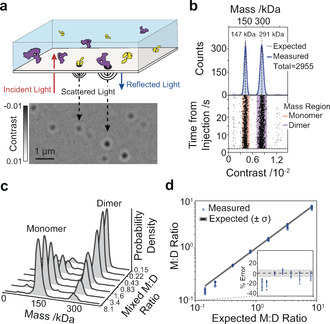
Principle of single‐molecule counting by mass photometry. a) Label‐free single‐molecule detection by imaging the interference of scattered and reflected light arising from individual protein landing events at a glass‐water interface over time. b) Scatter plot of single‐molecule contrasts and resulting mass distribution for a 1:1 monomer/dimer 2G12 mixture. c) Mass distributions for varying 2G12 monomer/dimer ratios. d) Comparison of monomer/dimer ratios measured by MP compared to expectations based on UV‐VIS absorption characterization.

Label‐free single‐molecule detection in principle provides the purest and most direct measurement of sample concentration by counting individual molecules. To explore this capability in the context of biomolecules, we chose monomers and domain exchanged dimers of the HIV‐1 neutralizing antibody 2G12 (see Figures S1–S3 in the Supporting Information), which produced mass distributions with the expected major bands at 147 kDa and 291 kDa (Figure 1 b). Repeating these experiments for monomer/dimer ratios ranging from 0.15 to 8.1 (Figure 1 c) revealed close agreement with UV‐VIS‐based characterization within the experimental error (4.6 % RMS), except for noticeable deviations (≈20 %) for the lowest ratios (Figure 1 d). We found that such deviations could almost exclusively be attributed to sample preparation, such as an additional dilution step required to reach sub‐nanomolar concentrations, leading to variations in counts arising from nonspecific protein adsorption to the sample tube (see Figures S4 and S5).

Equipped with these benchmarking results, we set out to investigate the suitability of MP to characterize interactions of varying affinities, using the immunoglobulin G (IgG) monoclonal antibody trastuzumab (Herceptin®) binding to soluble domains of IgG Fc receptors or ErbB2 (HER2) antigens. Trastuzumab, herein referred to as IgG, and FcγRIa by themselves revealed monodisperse distributions at 154±1 kDa and 50±1 kDa, respectively (Figure 2 a, see Figure S6). A 1:1 FcγRIa‐IgG mixture resulted in a large IgG‐FcγRIa complex peak, corresponding to about a 90 % complex formation, from which we can extract an apparent *K*_d_=50±10 pm by counting bound and unbound species in combination with knowledge of the total protein concentration [see Equations (S1)—(S6) and Figure S7]. IgG N‐glycan removal (see Figure S8) weakened FcR binding^29^ resulting in a 1:1 mixture of FcγRIa and deglycosyated IgG exhibiting considerably less bound antibody (ca. 50 %; Figure 2 b), corresponding to an apparent *K*_d_=1.0±0.1 nm (see Figure S9).

**Figure 2:**
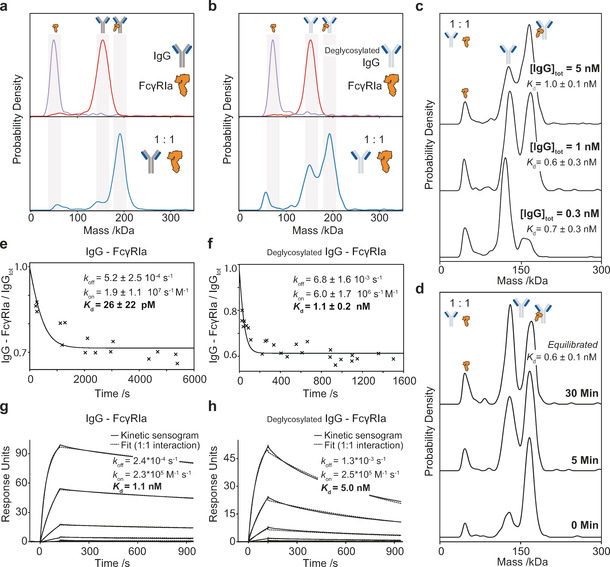
Single‐shot *K*_d_ and kinetics measurements of IgG‐FcγRIa interactions. a) MP mass distributions of IgG (red), FcγRIa (purple) and a 1:1 mixture of IgG‐FcγRIa (blue). b) MP distributions of deglycosylated IgG (red), FcγRIa (black) and 1:1 mixture of IgG‐FcγRIa (blue). c) Mass distributions for a 1:1 mixture of deglycosylated IgG‐FcγRIa at total IgG concentrations ranging from 300 pm to 5 nm and respective *K*_d_ calculated from a single‐shot measurement. d) Mass distributions for a 1:1 mixture of deglycosylated IgG‐FcγRIa at 1.5 nm total IgG concentration, ranging from 0 to 30 minutes after dilution from 2.9 μm. e,f) Mole fraction of assembled IgG‐FcγRIa and deglycosylated IgG‐FcγRIa complexes as a function of time after dilution from 2.7 μm to 0.3 nm total IgG concentration, and 2.6 μm to 5 nm total deglycosylated IgG concentration and corresponding single exponential fits. g,h) Corresponding SPR analysis of IgG‐FcγRIa and deglycosylated IgG‐FcγRIa (h).

This simple single‐shot approach presented so far produces results in a few minutes, however, necessarily neglects the importance of kinetics and equilibration conditions. To address this, we probed FcγRIa binding to deglycosylated IgG at a 1:1 ratio. Samples were mixed at 4 μm concentrations, incubated for 15 minutes, and diluted to 5, 1, and 0.3 nm as final protein concentrations (Figure 2 c; see Figure S10). At concentrations above the *K*_d_ value we found mostly bound complexes, with free species dominating below the *K*_d_ value, but all measurements yielded similar binding affinities (*K*_d_=1.0±0.1, 0.6±0.1 and 0.7±0.3 nm), suggesting that they were performed at or close to equilibrium (see Figure S11). These binding affinities were confirmed after equilibration time screening (see Figures S12 and S13).

For quantification of the tighter interaction between FcγRIa and IgG, screening at a range of concentrations was essential to ensure that the observed mass distributions were representative of the interaction to be quantified (see Figures S14–S16). As an additional example, for the HER2–IgG interaction, a simple single‐shot experiment at nanomolar concentration would have led to *K*_d,1_=1.4±0.1 nm and *K*_d,2_=4.8±0.3 nm (see Figure S17). Recording distributions at a few different concentrations, however, revealed a linear dependence of our *K*_d_ values on sample concentration, indicating a very tight *K*_d_<70 pm, and/or slow interactions with off‐rates on the order of hours. Therefore, performing a few measurements at a range of concentrations is crucial to prevent misinterpreting data derived from a single‐shot *K*_d_ approach for very strong interactions. Irrespective, our method provides rapid and clear distinction between interactions with vastly different binding affinities, which only need to be refined if highly accurate measurements are required.

The importance of (dis)association rates in addition to thermodynamic quantities raises the question to which degree we can use MP to directly visualize and quantify interaction kinetics. As MP measurements currently take place in the <100 nm concentration range, we should be able to access dissociation kinetics by simply diluting to total protein concentrations around the estimated *K*_d_ (approx. 1:1 ratio bound: unbound species for a 1:1 interaction), and monitoring the bound/unbound ratio throughout (Figure 2 f, see Figure S18 a). The observed exponential decay reveals the desired kinetic information, while the plateau yields the *K*_d_ value, ultimately enabling us to determine *k*_off_ and *k*_on_. For FcγRIa binding to deglycosylated IgG, this approach yielded *K*_d_=1.1±0.2 nm in good agreement with our single‐shot measurements (Figure 2 c), with *k*_on_=6.0±1.7×10^6^ m ^−1^ s^−1^ and *k*_off_=6.8±1.6×10^−3^ s^−1^. The corresponding experiment with glycosylated IgG‐FcγRIa yielded *K*_d_=26±22 pm with an off‐rate one order of magnitude slower (5.2±2.5×10^−4^ s^−1^) than for deglycosylated IgG but an almost identical on‐rate (1.9±1.1×10^7^ m ^−1^ s^−1^; Figure 2 e), again in good agreement with our single‐shot screening data (see Figures S15 and S16). The difference in *K*_d_ values between the glycosylated and deglycosylated IgG originates mostly from the off‐rate caused by protein–protein interactions (see Figure S19), confirming that the glycans are critical for tight binding. Association measurements (see Figures S18 b, S20, and S21) can in principle be used in an analogous fashion, although we found it more susceptible to protein loss because of nonspecific adsorption (see Figures S22–S24). Overall, our results were in good agreement with SPR measurements (Figures 2 e,f), subject to on‐rate variations expected from a matrix and surface‐immobilization‐based approach compared to ours, where all interactions take place in free solution (see Figure S25).

A key advantage of MP over existing solution‐based approaches is our ability to distinguish directly between different species contributing to a multicomponent system, as given by the IgG:FcRn interaction involving as many as five different interacting species. FcRn regulates serum IgG half‐life and transcytosis to the fetus by a pH gradient in endosomes, yet the interplay between self‐assembly and IgG binding is disputed,^30^, ^31^ which are both important factors in biotherapeutic design. Based on the existing literature we based our calculations on the independent free monomer binding model (see Figure S26 a). At pH 5 FcRn formed monomers and dimers with a *K*_d_=31±11 nm (Figure 3 a; see Figure S27). At pH 5.5 and pH 6, only negligible amounts of FcRn dimers were present with a FcRn monomer‐dimer *K*_d_>200 nm (see Figures S28 and S29). The resulting *K*_d_ values for the IgG‐FcRn interaction, at pH 5, were 44±9 nm for the monomer‐dimer equilibrium, 59±8 nm for the IgG‐FcRn_monomer_, and 6.6±0.6 nm for IgG plus two FcRns [see Figure S30 and Equations (S7)–(S18)]. Increasing the pH value to 5.5 decreased the binding affinities to 171±19 nm and 225±20 nm but did not significantly affect the binding affinity of IgG plus two FcRns of 3.9±1.5 nm (Figure 3 b; see Figures S31 and S32), contrasting SPR results for similar systems reporting an ensemble *K*_d_=760±60 nm for all interactions.^31^ At pH 6 and 7, our current sensitivity only allowed an estimate of the binding affinities to be *K*_d_>200 nm (Figure 3 b; see Figures S33 and S34). These results highlight pH‐dependent FcRn dynamics and IgG engagement, and reveal cooperativity where the second receptor binds IgG tighter than the first and with a weaker pH sensitivity.

**Figure 3:**
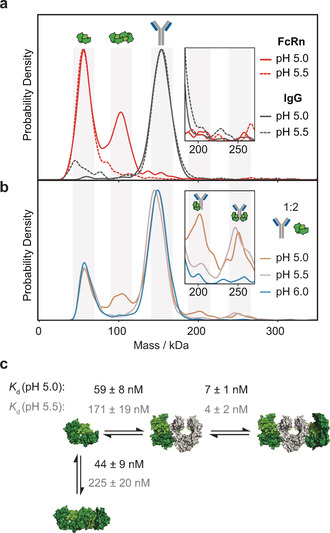
Binding stoichiometry and affinity of the IgG‐FcRn interaction as a function of pH. a) Self‐assembly of FcRn dimers at pH 5 (red) and 5.5 (dotted red) and equivalent pH measurements of IgG at pH 5 (grey) and 5.5 (dotted grey). b) IgG‐FcRn complexes (1:1 mixture) at pH 5, 5.5 and 6. c) Associated pH dependent binding affinities of interaction revealing cooperativity in FcRn binding (PDB: 4N0U, 3FRU).

Taken together, we have demonstrated that molecular counting with MP is sensitive, quantitative, and accurate in determining the relative abundances of different biomolecules and their complexes in solution. When implemented in the vicinity of the binding affinity, a single measurement lasting typically 30 seconds, or 240 seconds for continuous flow injection, yields accurate binding affinities spanning four orders of magnitude from 30 pm to 200 nm, while enabling kinetic probing with a time‐resolution on the order of 30 seconds in the range of minutes to hours. As a result, MP affords real‐time assessment of (dis)assembly completely label‐free and independent of protein immobilization to a surface, thus minimizing any possible perturbations, as well as being intrinsically sensitive to binding stoichiometries and oligomerization. The current limitation to sub‐micromolar affinities and concentration range can be addressed in the future through combination with fluidic approaches,^32^ as well as improvements to hardware and software, with which we expect to reach the micromolar range in the future. This range will enable measurements up to 100 μm affinities, making MP a powerful approach for characterizing biomolecular interactions without labels and single‐molecule sensitivity in a minimally perturbative fashion. Furthermore, the applicability of MP to both nucleic acids^33^ and large multimolecular machines^34^ provides scope for MP becoming a universal tool for studying biomolecular interactions and dynamics in a rapid, label‐free, yet single‐molecule‐sensitive fashion.

## Experimental Section

Protein preparation, mass photometry, data analysis as well as supplementary figures and equations are described in the Supporting Information.

## Conflict of interest

P.K. is an academic founder, shareholder and director to Refeyn Ltd. J.L.P.B. is an academic founder, shareholder and consultant to Refeyn LTd. W.B.S. is a shareholder and consultant to Refeyn Ltd. All other authors declare no conflict of interest.

## Supporting information

As a service to our authors and readers, this journal provides supporting information supplied by the authors. Such materials are peer reviewed and may be re‐organized for online delivery, but are not copy‐edited or typeset. Technical support issues arising from supporting information (other than missing files) should be addressed to the authors.

Supplementary
