# Supplementary material for: Quantifying Protein–Protein Interactions by Molecular Counting with Mass Photometry
Source: Angew Chem Int Ed Engl. 2020 Apr 2;59(27):10774–9. doi: 10.1002/anie.202001578 (PMC7318626; doi:10.1002/anie.202001578)
Supplement: Supplementary file 1 — Supplementary [file ANIE-59-10774-s001.pdf]

Angewandte Chemie
Eine Zeitschrift der Gesellschaft Deutscher Chemiker

Supporting Information

Quantifying Protein–Protein Interactions by Molecular Counting with Mass Photometry

Fabian Soltermann, Eric D. B. Foley, Veronica Pagnoni, Martin Galpin, Justin L. P. Benesch, Philipp Kukura, and Weston B. Struwe**

anie_202001578_sm_miscellaneous_information.pdf

Author Contributions

Conceptualization: F.S., P.K. and W.B.S.; Investigation: F.S., E.F. and V.P.; Formal Analysis: F.S. and M.G.; Writing—Original Draft: F.S., P.K., and W.B.S.; Writing—Review & Editing: all authors; Visualization: F.S., J.L.P.B. and W.B.S.; Supervision: P.K. and W.B.S.

1. Synopsis 4
2. Materials 4
3. Sample Preparation 4
3.1 Relative abundance measurements 5
3.2 Supplementary Table 1: 2G12 stock solutions and diluted concentrations. 5
3.3 Screening Experiments 5
3.4 Supplementary Table 2: FcR/IgG mixtures and incubation times 6
3.5 ErbB2-trastuzumab interaction study 6
4. Mass Photometry 6
4.1 Quality Control for Quantitative Measurements 7
4.2 Supplementary Table 3: Quality Data Criteria & Indicators 7
5. Data Analysis 8
5.1 Supplementary Table 4: Total Number of Counts for Kinetic Experiments 9
6. Protein Passivation of Sample Tubes 9
7. Non-specific Protein Adsorption to Sample Tubes 10
8. Surface Plasmon Resonance 10
9. Native Mass Spectrometry 11
10. Supplementary Figures 12
Supplementary Figure 1: Size exclusion chromatography of 2G12. 12
Supplementary Figure 2: SDS-PAGE of SEC purified 2G12. 13
Supplementary Figure 3: Native mass spectrometry of 2G12 and contrast calibration. 14
Supplementary Figure 4: Dynamic range estimation, sample carriers and diffusion correction. 15
Supplementary Figure 5: Data analysis approaches for 2G12 monomer/dimer ratios using gaskets. 16
Supplementary Figure 6: Technical replicates of purity screening of trastuzumab and FcγRIa. 17
Supplementary Figure 7: Technical replicates of IgG-FcγIa binding. 18
Supplementary Figure 8: Native MS to confirm IgG deglycosylation. 19
Supplementary Figure 10: Screening method for quantifying binding affinities and kinetics with MP. 21
Supplementary Figure 11: Concentration-dependent K_d distribution for $IgG_{deglycosylated}$-FcγIa. 22
Supplementary Figure 12: Equilibration time screening and assignment of FcγRIa molecular mass. 23
Supplementary Figure 13: Equilibration time screening of K_d for $IgG_{deglycosylated}$-FcγIa. 24
Supplementary Figure 14: Concentration screening of IgG-FcγIa complexes. 25
Supplementary Figure 15: Equilibration time screening for IgG-FcγIa. 26
Supplementary Figure 16: K_d values from equilibration time screening for IgG-FcγIa. 27
Supplementary Figure 17: Screening the trastuzumab-HER2 interaction. 28
Supplementary Figure 18: Principle of dissociation and association measurements. 29
Supplementary Figure 19: Correlation of published K_d vs k_{off} values. 30

Supplementary Figure 20: Association measurements (k_{on}) of $IgG_{deglycosylated}$-FcγIa. 31
Supplementary Figure 21: Association measurements (k_{on}) of IgG-FcγIa...32
Supplementary Figure 22: Non-specific IgG adsorption to samples tubes...33
Supplementary Figure 23: Protein passivation of sample tubes with BSA...34
Supplementary Figure 24: Protein passivation of sample tubes with casein..35
Supplementary Figure 25: Schematic of interactions in MP and SPR. 36
Supplementary Figure 26: Proposed binding models for IgG-FcRn interactions. 37
Supplementary Figure 27: Technical replicates of IgG and FcRn pH = 5. 38
Supplementary Figure 28: Technical replicates of IgG and FcRn pH = 5.5. 39
Supplementary Figure 29: Technical replicates of IgG and FcRn pH = 6.0. 40
Supplementary Figure 30: Time point and K_d measurements of IgG-FcRn at pH = 5.............................41
Supplementary Figure 31: Time point and K_d measurements of IgG-FcRn at pH = 5.5..........................42
Supplementary Figure 32: Time course measurements of IgG-FcRn binding at pH = 5.5......................43
Supplementary Figure 33: Time course measurements of IgG-FcRn binding at pH = 6.0......................44
Supplementary Figure 34: Time course measurements of IgG-FcRn binding at pH = 7.0......................45
11. Supplementary Table 5: Raw data of published biomolecular binding affinities................................46
12. Equations...49
References 52
Author Contributions...52

1. Synopsis

We first characterised all compounds separetely by MP to assess their oligomeric composition (**Supplementary Figure 10**). Subsequently, we prepared stoichiometry-dependent mixtures of analyte and binding partner (1:1 or 1:2) at µM concentration and equilibrated at room temperature overnight. The samples were then diluted to concentrations near the expected K_d value and equilibrated during a time *t* before conducting a concentration screening (**Supplementary Figure 10**). In these experiments we set the equilibration time to *t* = 15 min and measured the sample at three different dilutions (0.3 nM, 1 nM, 5-50 nM) to obtain an estimate of the K_d and, most importantly, to confirm that complexes are dissociating upon dilution, i.e. the signal corresponding to the biomolecular complex decreases in relative intensity (see **Figure 2c & 2d**). If complexes do not dissociate upon dilution, i.e. relative peak intensities are not changing, the biomolecule interaction is likely to exhibit very tight binding affinities (K_d < 0.1 nM) and/or slow kinetics (> 5 hrs) (**Supplementary Figure 15**) possibly beyond the dynamic range and sensitivity of the current MP instrumentation (**Supplementary Figure 17)**. Provided the K_d screening was successful we proceeded with the kinetic studies for which we diluted the sample to a concentration around the estimated K_d. After dilution/mixing we followed dissociation/association of the biomolecular complex by acquiring individual MP measurements at different time points. From these experiments, we obtained K_d values and on-and off-rates. With the current dynamic range we can inject concentrations from 100 pM to 50 nM, and thus determine quantitate binding affinities ranging from 10 pM to 300 nM and measure kinetics ranging from a few minutes to several hours.

2. Materials

The 2G12 antibody was expressed and purified as described previously[1]. Briefly, Plasmids encoding 2G12 antibody heavy and light chains were transiently expressed in HEK 293F at a cell density of 1 × 10^6 cells/mL with a 1:2 construct ratio (heavy to light chain). After 5 days, cells were pelleted and the supernatant was loaded onto a HiTrap Protein A column (GE Healthcare) following the manufacturers protocol. The binding buffer was 20 mM sodium phosphate, pH 7.5, and the elution buffer was 0.1 M citric acid, pH 3. Antibodies were immediately neutralized with 1 M Tris-HCl, pH 9, prior to buffer exchange into phosphate buffered saline (PBS) (Gibco® DPBS) using a 50 kDa cut-off spin filter (Vivaspin, Sartorius). Antibody oligomers were then separated with size exclusion chromatography (HiLoad Superdex 16/600 200 ps, GE Healthcare) and fractions were collected according to the SEC profile and SDS-PAGE (NuPAGE™ 4-12% Bis-Tris Protein Gels, Invitrogen™). Antibodies were stored at 4°C. Trastuzumab (Herceptin®) was purchased from Oxford University Hospital, CD64 human recombinant (His Tag) was purchased from SinoBiological, recombinant human FcRN (His and Avi tagged) was purchased from Stratech Scientific Ltd, ErbB2 (Her2) human recombinant was purchased from Sigma-Aldrich. Deglycosylated trastuzumab was obtained by treatment with Endo S (New England BioLabs) at 37 °C, overnight. 2G12 antibody was used for contrast vs. molecular weight calibration, with monomer and dimer masses of 148.5 and 296.9 kDa respectively (**Supplementary Figure 3**).

3. Sample Preparation

Samples and buffers were filtered through 0.1 µm centrifugal filters (Ultrafree –MC- VV, MerckMillipore) prior to use. Initial stock concentrations of all proteins were measured using a DeNovix DS-11+ spectrophotometer. Protein solutions were diluted in PBS (Gibco

DPBS (1X)) to obtain concentrations between 0.2 to 1 absorbance at 280 nm (low µM range). Pipetting precision was checked with a microbalance (Mettler Toledo, AT261).

3.1 Relative abundance measurements

For the experiments determining the accuracy of determining relative abundances (**Figure 1a-d**), we used the two SEC-fractions of the 2G12 antibody purification (**Supplementary Figure 1**), containing either the pure monomer or the intermolecular domain exchanged dimer version of the monomer[2]. Characterizion of the monomer fraction with MP confirmed the absence of dimerization and analogously the dimer fraction revealed absence of dissociation into monomer. Different ratios of the two non-interacting species (monomer and dimer) were mixed at µM concentration and diluted in PBS to pM-nM concentrations prior to measurement (**Supplementary Table 1**). For the two lowest M:D ratios (0.15 and 0.22) in **Figure 1c & 1d** the pure monomer stock solution had to undergo an additional step from 1 µM to 500 nM. All pipetting steps were checked on a microbalance.

3.2 Supplementary Table 1: 2G12 stock solutions and diluted concentrations.

Figure	2G12 monomer (stock mixture) /µM	2G12 dimer (stock mixture) /µM	2G12 monomer (diluted mixture) /nM	2G12 dimer (diluted mixture) /nM
1 a	0.59	0.74	2.9	3.6
1 b	0.59	0.74	2.9	3.6
1 c + d	1.4	0.17	5.3	0.63
	1.2	0.35	4.9	1.4
	0.88	0.55	3.9	2.4
	0.59	0.74	2.9	3.6
	0.37	0.88	2.0	4.8
	0.19	0.88	1.2	5.7
	0.14	0.95	0.90	6.1

3.3 Screening Experiments

Screening experiments (**Figure 2a-d and Figure 3**): Individual FcRs (FcγRIa, FcRn) and IgGs (trastuzumab and deglycosylated trastuzumab) were mixed at µM concentration in a 1:1 (IgG-FcγRIa, deglycosylated IgG-FcγRIa) or 1:2 (IgG-FcRn) ratio and incubated overnight at room temperature (see **Supplementary Table 2** for exact concentrations). The µM mixtures were diluted in PBS to pM-nM concentrations and measured at specific incubation times (**Supplementary Table 2**). For FcRn-IgG, this procedure was repeated for each pH (5.0, 5.5, 6.0, 7.2) (**Figure 3**). *Kinetic experiments* (**Figure 2e & 2f**)*:* Dissociation experiments were prepared as described for those shown in **Figure 2a-f** and repeated after specific incubation times. For association experiments, individual proteins were diluted in PBS to pM-nM concentrations and then mixed in 1:1 ratios. Measurements were taken after specific incubation times had elapsed. For association experiments of IgG and FcγRIa (shown in **Supplementary Figure 20 & 21**), the pure compounds were diluted

to nM concentrations, mixed, then incubated for the desired incubation time and then measured by MP. The sample tube passivation protocol, based on Casein was applied (**see below**).

3.4 Supplementary Table 2: FcR/IgG mixtures and incubation times

	Figure	FcR (stock) /µM	IgG stock) /µM	FcR (diluted mixture) /nM)	IgG (diluted mixture) /nM	incubation time after dilution /min
screening experiments	2 a	2.7	2.7	4.4	4.4	10-18
	2 b	2.9	2.9	4.6	4.6	12-18
	2 c	2.9	2.9	0.3, 1.3, 4.6	0.3, 1.3, 4.6	10-35
	2 d	2.9	1.3	1.3	1.3	0.4-30
	3a	18.8	7	11.8	9.2	0.4-89
	3b	16.9	7.6	0.4-6.0	0.2- 2.7	0.5-78
kinetic experiments	2e	2.7	2.7	0.21- 0.26	0.21- 0.26	1.8-88
	2f	2.6	2.6	4.6- 4.9	4.5- 4.8	0.3-58

3.5 ErbB2-trastuzumab interaction study

Experiments were performed analogously to IgG-FcγRIA. Briefly, we applied the K_d screening procedure to investigate the binding affinity of trastuzumab to the ErbB2 (HER2) antigen. The two compounds were mixed in a 1:1 molar ratio and incubated at room temperature overnight. The next day, MP data was acquired at different dilutions (0.3, 1.3, 4.6 nM trastuzumab concentration) and time points after dilution (2.5 – 100 min). Gaskets (see below) were used for the experiments with the 1.3 and 4.6 nM dilutions and the continuous flow setup for the 0.3 nM dilution (**Supplementary Figure 4**).

4. Mass Photometry

Microscope coverslip cleaning and assembly was performed as described previously[3]. Borosilicate microscope coverslips (No. 1.5H thickness, 24 x 50 mm, VWR (630-2603) were cleaned by 5 min sequential sonication (PS-40A Ultrasonic Cleaner, Cole Palmer) in Milli-Q water, isopropanol and Milli-Q water, followed by drying under a clean stream of nitrogen. Quality control of new batches of coverslips was based on checking the surface roughness on the mass photometry native images for 6 out of 100 coverslips from a single box. Batches with large particles or irregularities on the glass surface were disposed. Clean coverslips were assembled for sample delivery using silicone gaskets (CultureWell™ reusable gasket, 3mm diameter x 1 mm depth, Grace Bio-Labs) or plastic flow chambers (sticky-Slide VI 0.4, Ibidi). For experiments involving flow chambers, a microfluidic syringe pump-based system was used (World Precision Instruments AL-1000) (**Supplementary Figure 4**).

For data acquisition, gaskets were filled with buffer (40 µl) to enable focusing of the glass surface. Subsequently, 36 µl were removed, while keeping the focus position stable, and replaced by 36 µl sample solution at nM concentration. Measurements were started immediately (delay time ~2 seconds) for 30 - 60 seconds. For samples with sub-nM K_d values, we conducted continuous flow experiments at sub-nM concentrations typically over 240 s. Flow chambers were filled with 150 µl buffer and connected to the sample reservoir and syringe pump, while preventing the generation of air bubbles in the flow chamber. The flow chamber was then placed on

the instrument in a way that the laser was positioned close to the inlet of the flow chamber. Next, the syringe pump was started in pump mode and run at 90 µl /min. Once the sample reservoir was almost empty, the sample (sub-nM concentration) was injected, focus checked and the measurement started. The acquisition time was chosen depending on concentration, i.e. lowering the concentration by a factor of 10 requires 10 times longer acquisition times to keep the total number of molecular counts constant. Continuous flow experiments at concentrations in the nM range or higher should be avoided, because the coverslip glass surface becomes saturated with analyte molecules within 1 min after injection.

The experimental mass photometry setup has been described elsewhere[3,4]. Briefly, a 525 nm laser diode was used for illumination with the following instrument parameters: acquisition camera frame rate = 955 Hz, pixel binning = 4x4, 5-fold time-averaging. Data was acquired for 30 or 240 s, depending on the experiment. Particle detection and quantification was processed as described by Young *et al*. using custom software written in Python[3]. For each particle a time stamp, position and contrast value was obtained.

4.1 Quality Control for Quantitative Measurements

During acquisition each video was examined visually and excluded when one of the criteria listed in **Supplementary Table 3** was met. During data analysis we applied an xy-distance constraint filter to all confirmed counts, excluding particles which were recorded at the same xy-position in the field-of-view. This helped to exclude “blinkers” (i.e. fast binding and unbinding events at the same position) and significantly lowered the baseline/noise. For gasket experiments, we plotted the counts as a detection rate (−∆counts/∆t) of the measured species X vs. time. The resulting decay curve was exected to follow a single exponential decay, which indicates an error-free experiment. We experienced deviations from a smooth decay when one or several of the criteria in **Supplementary Table 3** were given.

4.2 Supplementary Table 3: Quality Data Criteria & Indicators

(i.e. low molecular weight resolution and high noise level)

Criteria	Indicators	Most common reasons
Out-of-focus measurement	Inversed contrast or anomalous PSF of particles in ratiometric image or no counts	Wrong focus position
Unstable focus	Focus voltage is drifting	Excessive immersion oil between stage and coverslip
Rough/irregular glass surface	Large interfering black dots in native image	Insufficient quality control during glass cleaning procedure
Unbinding events	Particles with negative contrast values	Unsuitable pH, unsuitable glass surface properties (test APTES coating)[5]
Buffer impurities	Large particles floating around and causing interferences in the ratiometric image	Buffer or sample not filtered suitably (i.e. 0.22 µm filter), sample is precipitating under chosen conditions
Too high concentration	Particles are overlapping in time and position	Incorrect dilution

5. Data Analysis

Quantification of species-specific counts: Contrast values of observed counts were converted into molecular weight via a mass calibration (**Supplementary Figure 3**), here with the 2G12 WT antibody monomer and dimer peaks. In the resulting molecular weight (MW) vs. counts histogram each species was identified as a resolved peak and counts were obtained from Gaussian fitting to these peaks. Molecular weights near the MP detection limit (ca. 40 kDa) were not quantified to ensure differentiation from background noise and were generally not treated as quantitative with respect to counts for larger species (**Supplementary Figure 12**). The measured counts were then converted into molar concentrations following the protocol described in equations **1-5** (for IgG and FcγRIa) and **equations 7-13** (for IgG and FcRn). For continuous flow experiments, the counts were diffusion-corrected by normalising to the MW-dependent factor of the diffusion coefficient ($MW^{-1/3}$). For gasket experiments, the contribution from diffusion was negligible, as shown in **Supplementary Figure 4 & 5.**

K_d screening experiments **(Figure 2a-d and Figure 3a-c)**: K_d values are calculated by inserting the molar concentrations obtained from the previous section into **equation 6** for IgG-FcγRIa and **equations 14 -18** for IgG-FcRn.

Kinetic experiments **(Figure 2e & 2f, Supplementary Figures 20 & 21)**: Monitoring association or dissociation of the biomolecular complex allowed us to extract on-and off-rates. Additionally, we could extract K_d values after reaching equilibrium (plateau regions in the kinetic plots). Kinetic data sets were always composed of a series of individual MP experiments acquired at different time points after dilution from µM to pM-nM (continuous flow or gasket experiments). In both cases, we can extract the time-dependent ratio of free vs. bound species from each measurement at time *t* (e.g. **Figure 2e & 2f**). *Association experiment:* We begin by defining the differential equation describing the time-dependence for association (**equation 22-28**), based on the stoichiometric information from our MP experiments. In the association experiment, the starting concentrations of each species at *t* = 0 are known (**equations 19-21)**. We also know that $[IgG_{bound}]_0 = 0$ and $[IgG_{bound}] = x_t$, i.e. x_t is the amount of generated complex since mixing. The ordinary differential equation (ODE), **equation 28**, is numerically solved in Mathematica (Mathematica, Version 12.0) with the built-in function “Dsolve“ to obtain x_t as a function of k_{on} and k_{off}. The result for x_t is fit to the experimentally obtained ratio of the counts of IgG_{bound} and the total counts of IgG as described in **equation 29**. For fitting we used Mathematica’s built-in function “NonlinearModelFit” with the method “NMinimize” to optimize k_{on} and k_{off}. From this we obtain K_d, k_{on} and k_{off} values and confidence intervals (**Supplementary Figures 20 & 21).** Specifically, fitting involved two parameters (k_{on} and k_{off}), the NonlinearModelFit function returned 95% confidence intervals for the separate estimates of k_{on} and k_{off}. As these values were not symmetric about the mean, the reported error bars in **Figures 2e & 2f, Supplementary Figures 20 & 21** were the greater of the two values. This function also returned a 95% parameter confidence region in the “k_{on}, k_{off}” plane. This was used to calculate the largest and smallest $K_d = k_{off}/k_{on}$ that was within the confidence region. Similarly as above, these values were not symmetric about the mean and the greater of the two were reported. Scattering of the data was partly attributed to experimental error arising mostly from UV-VIS spectrophotometer concentration measurements, wtih an expected relative error of ~5%. The shot noise contribution to the scattering can be estimated from the mean relative standard deviation for each figure (3.8 ± 0.3%, 3.9 ± 0.4%, 5.1 ± 0.5% and 6.1 ± 0.7%) calculated from the total counts shown in **Supplementary Table 4**. *Dissociation experiment*: First, we calculate the K_d from the plateau region of the plot (**equations 1-6**). Knowing the K_d and the total concentrations

of IgG and FcγIa before dilution (ca. µM), we can calculate the equilibrium concentrations of each species before dilution by solving **equation 32** and inserting into **equations 30 & 31**. At t = 0 after dilution, this distribution is unchanged, and the concentrations have to be divided by the dilution factor **(equations 33-35)**. This yields $[IgG_{bound}]_0 = \frac{[IgG_{bound}]_{eq,conc}}{f_{dilution}}$ and $[IgG_{bound}] = [IgG_{bound}]_0 - x_t$, i.e. x_t is the amount of dissociated complex since diluting from the stock concentration to pM/nM concentration. Proceeding as described for the association experiment and using **equations 36-42** we obtain K_d, k_{on} and k_{off} values (**Figure 2e & 2f).** In the future, we could combine the information from association and dissociation data and perform the fitting iteratively/simultaneously, from which k_{off}, k_{on} and K_{d} can be obtained without waiting for equilibriation. This approach will extend our applicability to slow reactions by circumventing equilibration times of hours or even days (**Supplementary Figure 19**) and with this also reduce sample loss due to non-specific adsorption to sample tubes over time (**Supplementary Figure 22-24**).

5.1 Supplementary Table 4: Total Number of Counts for Kinetic Experiments

**Figure 2e**		**Figure 2f**		**Supplementary Fig. 20**		**Supplementary Fig. 21**	
Time after dilution / s	Counts	Time after dilution / s	Counts	Time after dilution / s	Counts	Time after dilution / s	Counts
222.5	688	26	927	32	409	28	518
259.8	570	28	611	36	396	53	404
265.3	818	39	820	43	396	70	370
1117.6	657	49	810	51	361	182	268
1180.8	546	69	647	66	264	210	404
1288.4	494	88	770	81	337	227	365
2029.1	849	112	463	116	258	352	403
2110	708	223	480	214	319	367	504
2400	738	253	502	244	261	508	364
2958.8	835	267	868	251	193	535	483
3046.1	573	336	564	322	251	560	464
4352.2	681	406	620	355	400	675	398
4675	715	467	697	368	309	685	464
5332.5	651	555	468	437	242	691	358
5389.9	800	611	894	441	220	720	416
		663	700	467	277	842	246
		884	712	505	275		
		888	786	635	245		
		937	717	659	253		
		990	760	687	200		
		1097	672	701	346		
		1100	673	824	263		
		1149	605	856	168		
		1356	712	903	305		
		1501	548	1042	245		
				1130	227		
				1457	236		

6. Protein Passivation of Sample Tubes

Bovine serum albumin (BSA, Sigma-Aldrich) was prepared as a 1 μM solution in phosphate buffered saline (PBS) (Gibco® DPBS). 1 ml of this solution was added to each sample vial (1.5 ml Safe-Lock Tubes, Eppendorf) and rotated for ca. 2 hrs at room temperature (VWR rotator, VWR). The solution was completely removed, washed 3 times with PBS (1 ml each), filled with PBS and rotated for 30 min, then washed 3 times with PBS (1 ml each). All liquid was removed and replaced by either PBS buffer or 5 nM trastuzumab

(Herceptin®). The trastuzumab solution was directly diluted in the passivated tube by mixing 2 μl of trastuzumab with 398 μl PBS. The control sample was prepared in the same way, but using an untreated sample vial.

Casein from bovine milk (technical grade, Sigma-Aldrich) was dissolved as 1 mg/ml in phosphate buffered saline (PBS) (Gibco® DPBS) at 42 C overnight. 1 ml of the solution was added to each sample vial (1.5 ml Safe-Lock Tubes, Eppendorf) and rotated for ca. 2 hrs at room temperature. The solution was completely removed, washed 3 times with PBS (1 ml each), filled with PBS and rotated for 30 min, then washed 3 times with PBS (1 ml each). All liquid was removed and replaced by either PBS buffer or 5 nM trastuzumab (Herceptin®). The trastuzumab solution was directly diluted in the passivated tube by mixing 2 μl of trastuzumab with 398 μl PBS. The control sample was prepared in the same way, but using an untreated sample vial.

7. Non-specific Protein Adsorption to Sample Tubes

*m*PEG-silane coated glass vials (1.75 ml, Samco Trident Vial Tall with PP Screw Cap) were sonicated (PS-40A Ultrasonic Cleaner, Cole Palmer) for 10 min in 2% aqueous Hellmanex solution (Hellmanex III, HellmaAnalytics), washed with deionized water, then sonicated for 10 min in deionized water. The sonication step was repeated in ethanol. In a next step the glass vials were thoroughly dried under a stream of dry nitrogen and then placed in an O_2 plasma (Zepto System, Diener Plasma Surface Technology) for 8 min. After plasma cleaning the vials were filled with a 5 mg/ml mPEG-silane solution in 1% acetic acid in ethanol. mPEG-silane was purchased from Laysan Bio as "MPEG-SIL-2000". The vials were then placed in an oven (Vacucenter, SalvisLab) at 70 °C and the reaction solution mixed after 30, 60 and 90 min. After 2 hrs, the vials were emptied, washed with ethanol and deionized water and blow-dried under a stream of dry nitrogen. *Plasma cleaned glass vials*: The protocol for "*m*PEG-silane coated glass vials" was stopped after the plasma cleaning step. *Tween treated glass vials* (1.75 ml, Samco Trident Vial Tall with PP Screw Cap) were incubated for 4 hours with 2 % aqueous Tween solution (Tween20, Sigma-Aldrich), then rinsed with deion. water and blow-dried under a stream of dry nitrogen. For *untreated glass vials* we used 1.75 ml, Samco Trident Vial Tall with PP Screw Cap. Forprotein LoBind tubes we used Protein LoBind Tubes (1.5 ml, Eppendorf). Safe-Lock Tubes (1.5 ml, Eppendorf) were used as Eppendorf tubes. *Tween treated Protein LoBind* tubes (Protein LoBind Tubes (1.5 ml, Eppendorf)) were incubated for 4 hours with 2% aqueous Tween solution (Tween20, Sigma-Aldrich), then rinsed with deionized water and blow-dried under a stream of dry nitrogen. *PCR tubes* were Fisherbrand 0.2 ml PCR tubes.

In the last step, all tubes were washed with phosphate buffered saline (PBS) (Gibco® DPBS), dried and then incubated with 5 nM trastuzumab (Herceptin®) in PBS for 20 hrs at room temperature. A freshly prepared 5 nM IgG solution in LoBind tubes was used as a reference. The acquisition time of MP experiments was 90 s. Absolute count values of the IgG peak were then used to compare the performance of different sample tubes, i.e. counts were used as an indicator for absolute concentration.

8. Surface Plasmon Resonance

Surface plasmon resonance experiments were carried out using a BIAcore T200 instrument (GE Healthcare). All experiments were performed in 10 mM HEPES, pH 7.4, 150 mM NaCl, 0.005% Tween 20 at 25 °C. Trastuzumab and deglycosylated trastuzumab mAb were immobilized on a CM5 chip (GE Healthcare) by amine coupling. Concentration series of human FcγRIa (A: 0.007-27 nM, B: 0.027-

110 nM) were flowed over IgG (trastuzumab) (A) or deglycosylated IgG (trastuzumab) (B) bound surface at 30 µl/min for 120 s followed by buffer for 900 s. After each run, the biosensor chip was regenerated using 10 mM glycine, pH 2.5, which breaks the antibody-FcγR interaction. The specific binding response to antibody was obtained by subtracting the response given by analytes to an uncoupled surface and a blank run of buffer only. The kinetic sensorgrams were fitted to a global 1:1 interaction model to allow calculation of k_{on}, k_{off}, and K_d using BIAevaluation software 2.0.3 (GE Healthcare).

9. Native Mass Spectrometry

Proteins were buffer exchanged into 1 M aqueous ammonium acetate (Sigma-Aldrich) using P6 Biospin columns (Bio-Rad) for the first two exchanges, followed by two exchanges using Amicon Ultra centrifugal filters (0.5 ml, 30 kDa MWCO) and in a last buffer exchange to 200 mM aqueous ammonium acetate using Amicon Ultra centrifugal filters. All protein solutions were analyzed at concentrations between 5 and 10 µM. Experiments were carried out on a prototype Thermo Scientific Q Exactive Hybrid Quadrupole Orbitrap Mass Spectrometer. Data were acquired using Xcalibur 3.0 software (Thermo Fisher Scientific) and raw spectra were deconvoluted to zero-charge spectra using UniDec[6].

10. Supplementary Figures

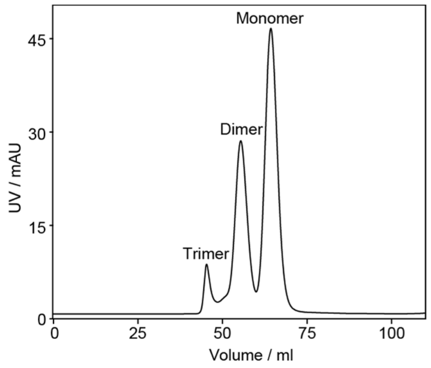

Supplementary Figure 1: Size exclusion chromatography of 2G12. UV profile of the monoclonal antibody 2G12 monomer, dimer and trimer complexes.

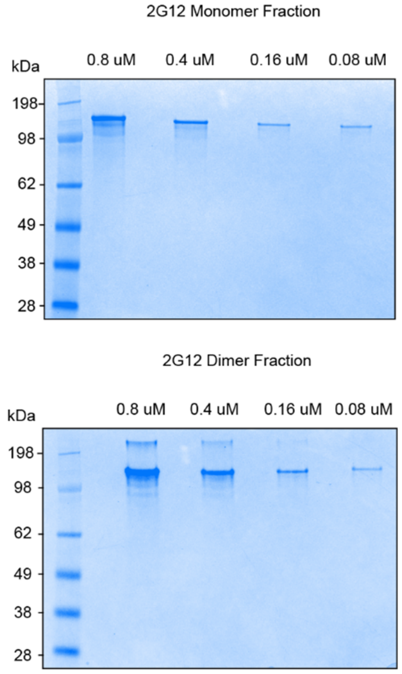

Supplementary Figure 2: SDS-PAGE of SEC purified 2G12. Monomers (top) and dimers (bottom). Concentrations from 0.8μM to 0.08 μM were imaged for each monomer/dimer SEC fraction.

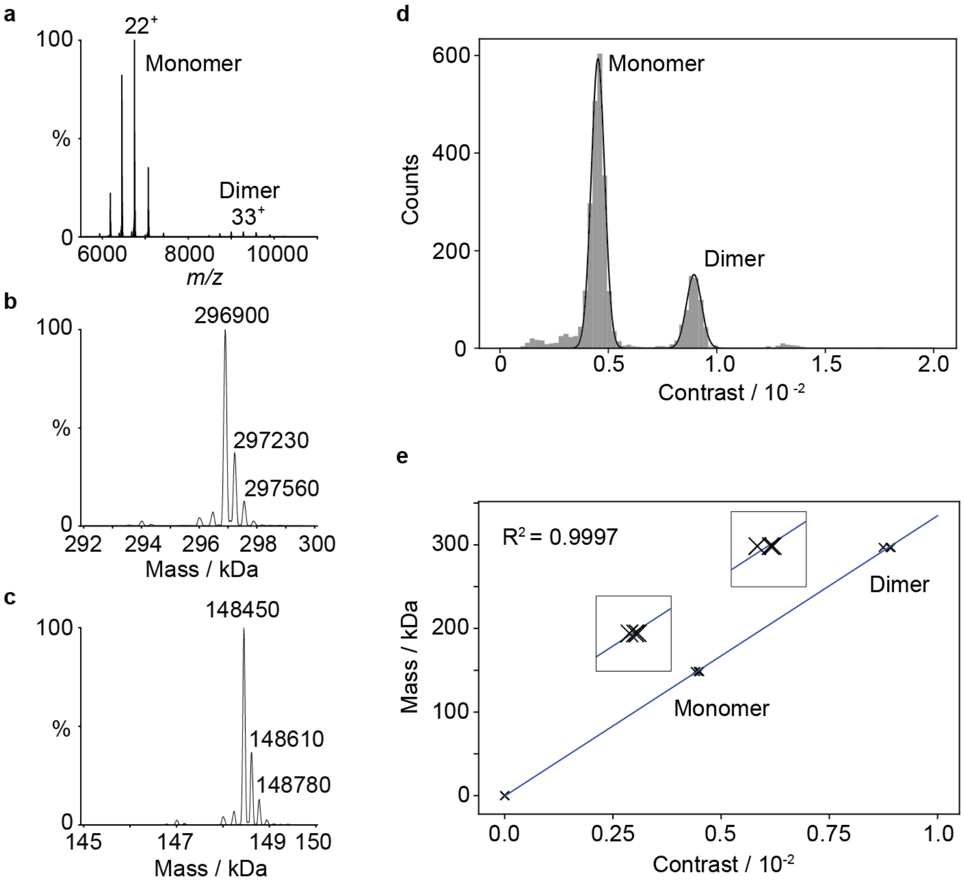

Supplementary Figure 3: Native mass spectrometry of 2G12 and contrast calibration. (**a**) Native mass spectrometry of 2G12 (top spectrum). (**b,c**) Zoom of the zero-charge state deconvoluted spectra, showing monomer mass range and dimer mass range. (**d**) Corresponding mass distribution obtained by MP. (**e**) MP mass calibration and reproducibility.

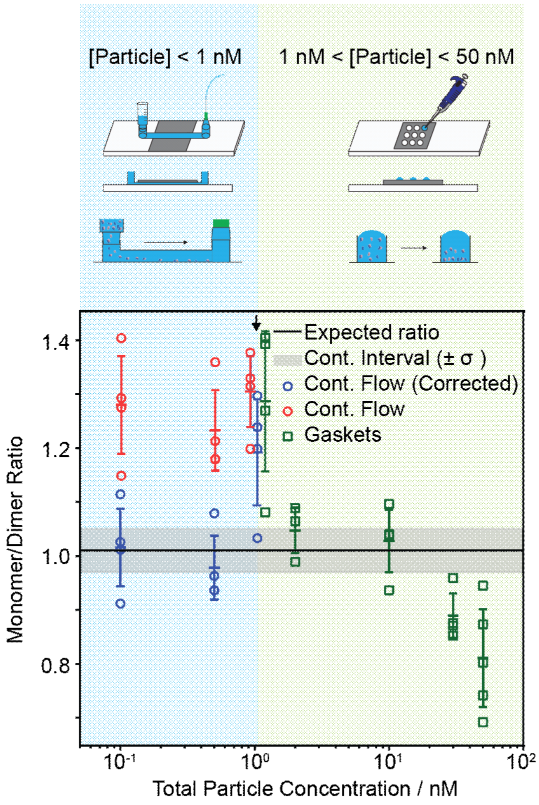

Supplementary Figure 4: Dynamic range estimation, sample carriers and diffusion correction. Experimental setup for continuous flow chambers (**top, left**) and silicone gaskets (**top, right**). MP ratio measurements of a 1:1 mixture of 2G12 monomer and dimer at different dilutions (0.1, 0.5, 1, 1, 2, 10, 30, 50 nM). The three results for 1 nM solutions were separated horizontally (**arrow**) to improve readability. Diffusion-corrected continuous flow ratios (blue) where obtained by normalizing monomer and dimer counts to their corresponding molecular weight dependent factor in the diffusion coefficient ($MW^{-1/3}$). The expected ratio (1.04 ± 0.05, black) was determined from UV-VIS measurements of concentrated (μM) stock solution of the pure monomer and dimer as well as weighing monomer and dimer stock solutions on a microbalance. These experiments were conducted to probe the dynamic range, suggesting that we can accurately measure ratios for samples with particle concentrations below 50 nM and extending the dynamic range to sub-nM concentrations when going from gaskets to a continuous-flow injection system. In the case of the continuous flow system we observed an influence of diffusion on our monomer:dimer ratios, which is expected to be molecular weight dependent ($MW^{-1/3}$). No influence was observed when conducting the experiments with gaskets when time between sample introduction and data recording was minimized (delay time <5 s) (**Supplementary Figure 5**).

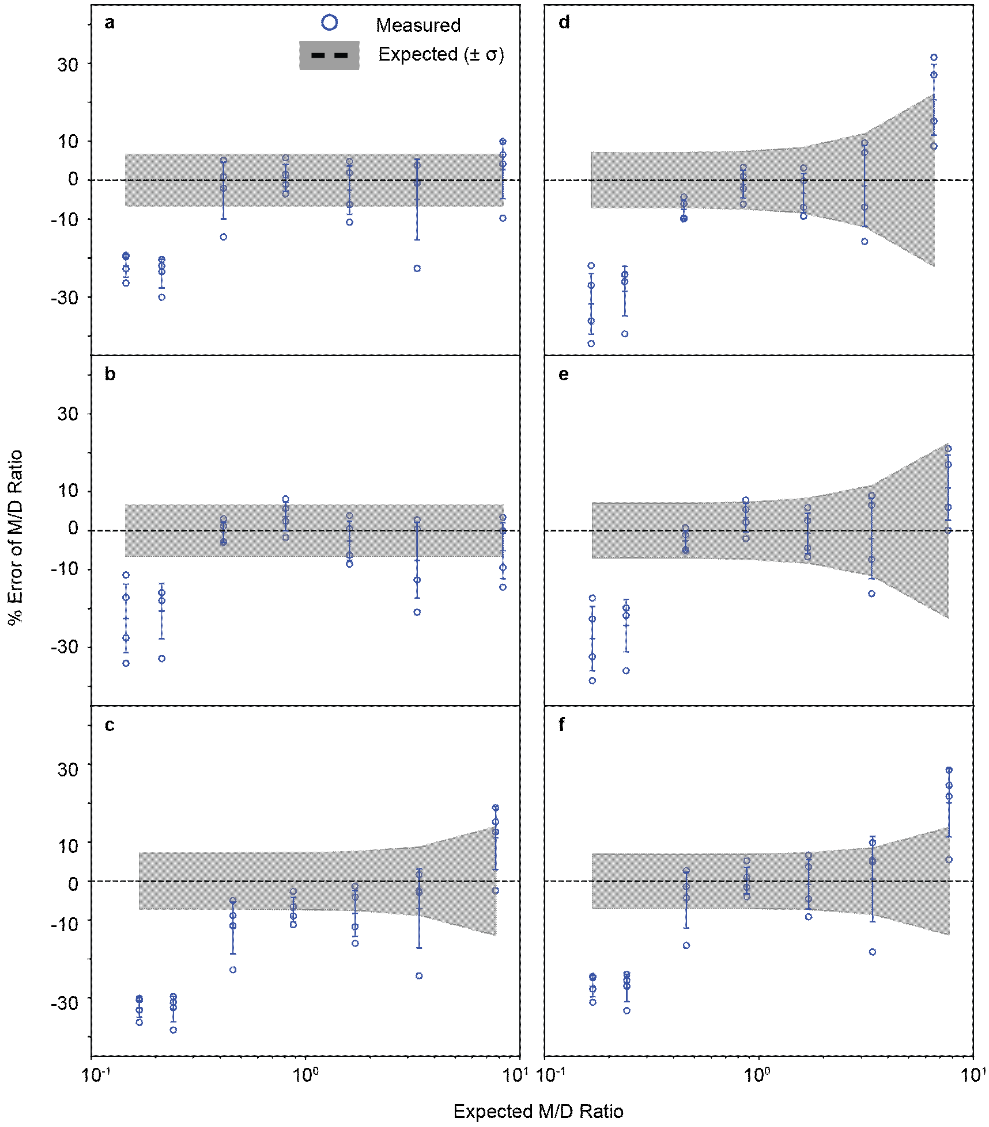

Supplementary Figure 5: Data analysis approaches for 2G12 monomer/dimer ratios using gaskets. We tested the influence of data acquisition duration, diffusion correction ($MW^{-1/3}$) and correcting for minor impurities. (**a**) No corrections, 90 s acquisition time. (**b**) No correction and 30 s acquisition time. The combination of accurate results, not needing to apply correction and having the highest time resolution were the main reasons to use method (**b**) in our work. (**c**) 90 s acquisition time and correction for minor impurities from control experiments of pure monomer, dimer and trimer. (**d**) 30 s acquisition time and correction for minor impurities from control experiments of pure monomer, dimer and trimer. (**e**) 90 s acquisition time and correction for minor impurities from control experiments of pure monomer, dimer and trimer and diffusion correction. (**f**) 30 s acquisition time and correction for minor impurities from control experiments of pure monomer, dimer and trimer and diffusion correction. We concluded that acquisition duration, diffusion correction and correction for minor impurities has only limited influence on the M:D ratio when the time between sample injection and acquisition start is kept below 5 seconds. From **Supplementary Figures 4 & 5**, we concluded that experimental procedures have to be carefully designed when working at sub-μM concentrations (e.g. when adding additional dilution steps) because of non-specific adsorption of protein to sample tube surfaces. Additionally, relative abundances obtained from continuous-flow injection should be diffusion-corrected. In the case of our injection procedure with gaskets, we show that sample diffusion, as well as minor small mass contaminants have minor effects on the 2G12 monomer:dimer ratio determined by molecular counting.

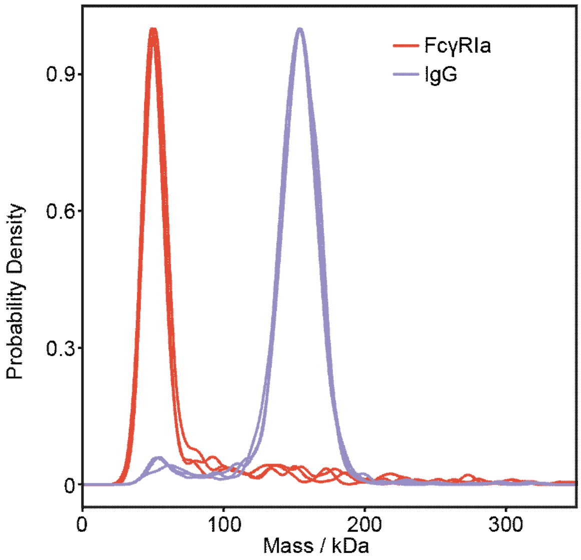

Supplementary Figure 6: Technical replicates of purity screening of trastuzumab and FcγRIa. Individual measurements of IgG (purple) were diluted from 7.4 µM to 5.2 nM and measured after 0.4, 3.8 and 7.6 min. Individual measurements of Fcγla (red) were diluted from 5 µM to 4.8 nM and measured after 0.4, 4.4 and 10.4 min. Small amounts of FcγRIa oligomers were visible, for IgG the data suggests high sample purity, with expected background at low molecular weight due to noise or fragments/residual impurities.

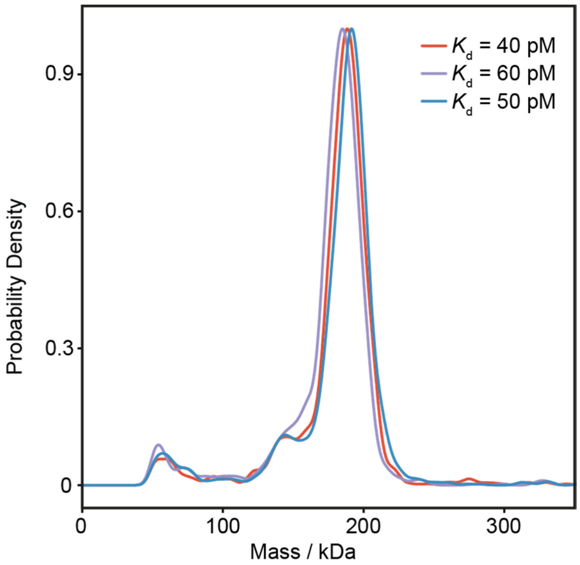

Supplementary Figure 7: Technical replicates of IgG-FcγIa binding. IgG-FcγIa were mixed 1:1 with final IgG concentration of 2.7 µM, followed by overnight incubation at room temperature. Samples were diluted to 4.4 nM IgG concentration and measured after 10, 14 and 18 min incubation time. Apparent K_ds in the low pM range were obtained. The small peak of free IgG at ca. 150 kDa could also originate from slighlty scewed 1:1 ratios due to uncertainity in the UV-VIS measurements.

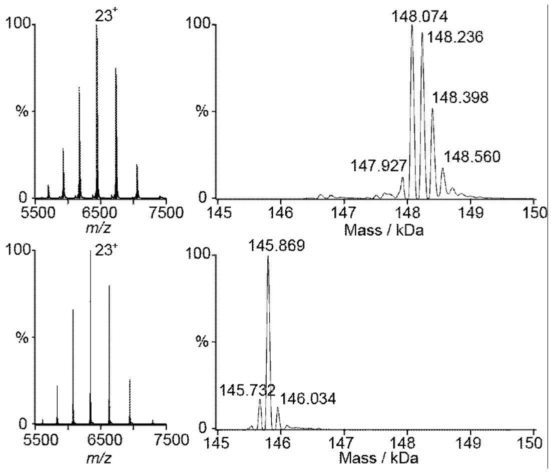

Supplementary Figure 8: Native MS to confirm IgG deglycosylation. Native MS of IgG (top, left) and corresponding zero-charge state deconvoluted spectrum (top, right). Corresponding deglycosylated IgG following treatment with Endoglycosidase S (native spectrum, bottom left and zero-charge state deconvoluted spectrum bottom, right).

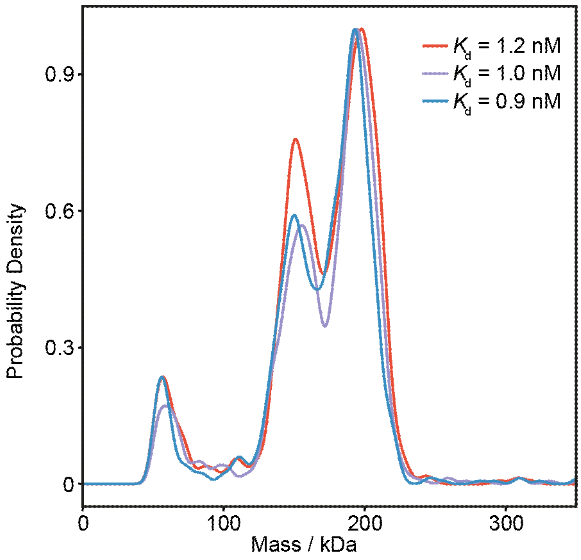

Supplementary Figure 9: Technical replicates of IgG$_{deglycosylated}$-FcγIa binding. IgG$_{deglycosylated}$ -FcγIa were mixed at a 1:1 ratio with final IgG concentration of 2.9 µM, followed by overnight incubation at room temperature. Samples were diluted to 4.6 nM IgG concentration and measured after 16, 13 and 18 min incubation time. Apparent K_ds in the low nM range were obtained.

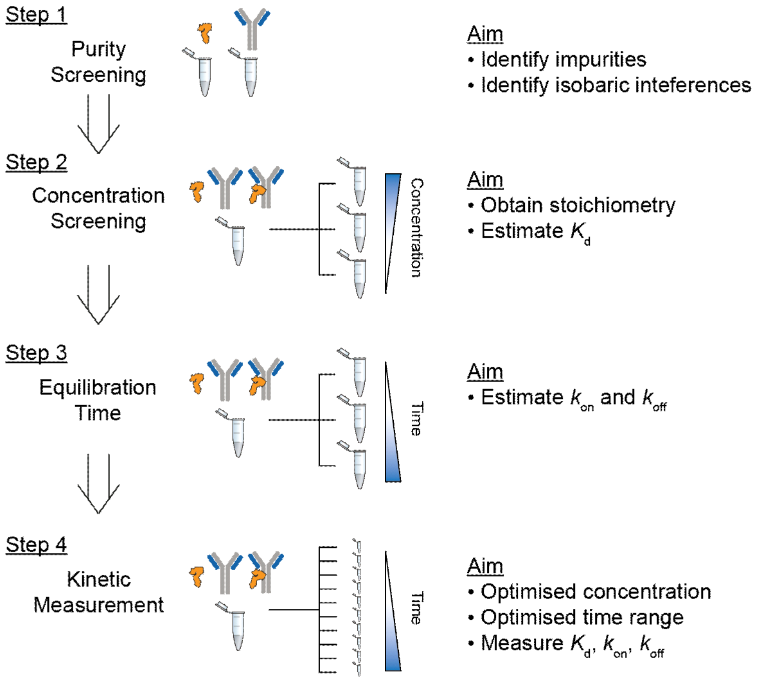

Supplementary Figure 10: Screening method for quantifying binding affinities and kinetics with MP. The ability to estimate binding affinities and kinetics in simple and fast screening experiments (concentration screening and equilibration time screening) offers a simple route to investigate a large number of candidates within a short time period and is crucial to prevent misinterpreting data derived from a single-shot K_d approach. This allows us to choose ideal experimental parameters, such as concentration range and equilibration time, for a time-resolved experiment with which we can accurately determine the on and off rates of the interaction (**Supplementary Figure 18**).

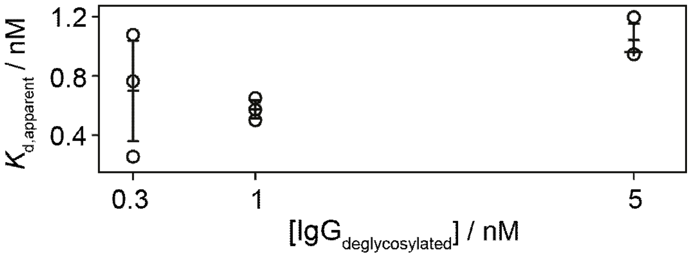

Supplementary Figure 11: Concentration-dependent K_d distribution for $IgG_{deglycosylated}$-FcγIa. $IgG_{deglycosylated}$-FcγIa were mixed at a 1:1 ratio with a final IgG concentration of 2.9 µM, followed by overnight incubation at room temperature. Samples were diluted (0.3, 1.3 or 4.6 nM IgG concentration) and measured for 10 - 34 min incubation time. The time dependence of the apparent K_d is best revealed for the 0.3 nM mixture (10 min at 0.3 nM: K_d= 0.25 nM, 22 min: K_d= 0.75 nM , 34 min: K_d= 1.1 nM, all at 0.3 nM).

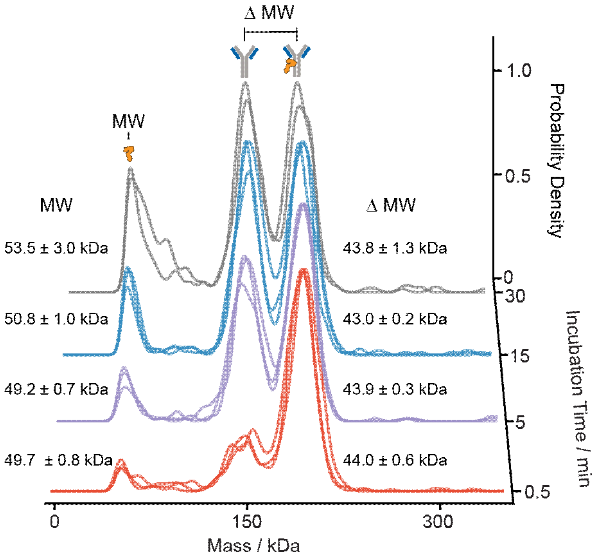

Supplementary Figure 12: Equilibration time screening and assignment of FcγRIa molecular mass. FcγRIa mass measured from the FcγRIa peak (left, around 50 kDa). FcγRIa mass measured from the mass difference between bound IgG (FcγRIa + IgG) and unbound IgG (right, 150- 200 kDa). We expect the molecular weight of FcγRIa (ca. 43-44 kDa) from the mass difference method to be more accurate than the direct read-out (49-54 kDa) due to its vicinity to the detection limit of our instrument. $IgG_{deglycosylated}$ -FcγIa were mixed at a 1:1 ratio with final IgG concentration of 2.9 µM, followed by overnight incubation at room temperature. Samples were diluted to 1.3 nM IgG concentration and measured after incubation times ranging from 0.4 - 30 min.

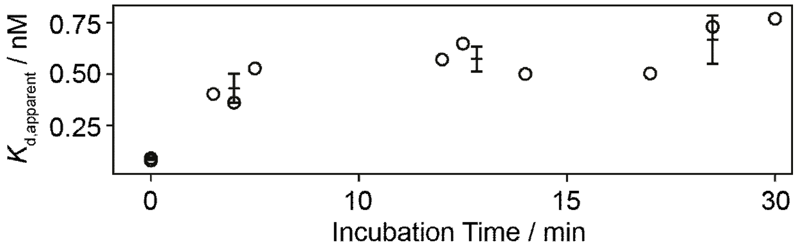

Supplementary Figure 13: Equilibration time screening of K_d for IgG$_{deglycosylated}$-FcγIa. For FcγRIa binding to deglycosylated IgG, shifts in peak intensities were observed during the concentration screening (**Supplementary Figure 10**), indicating that we should be able to observe the time dependence of these interactions. Measurements revealed that equilibrium was reached after 10 minutes, yielding K_d = 0.6 ± 0.1 nM (Figure 2d, Supplementary Figure 12). Repeating the same experiments (concentration and equilibration time screening) with FcγRIa-IgG highlighted the importance of this screening procedure (**Supplementary Figures 14-16**). For the here noted IgG$_{deglycosylated}$-FcγIa experiment we mixed the two compounds 1:1 with a final IgG concentration of 2.9 μM, followed by overnight incubation at room temperature. Samples were diluted to 1.3 nM and measured after 0.4-30 min incubation time (see **Supplementary Figure 12** for corresponding KDE-plots). Starting from apparent K_d values of 0.09 nM after 0.4 min incubation time we can observe a gradual increase over time, until we start to reach a plateau-region after ca. 10 min, suggesting a K_d = 0.6 ± 0.1 nM.

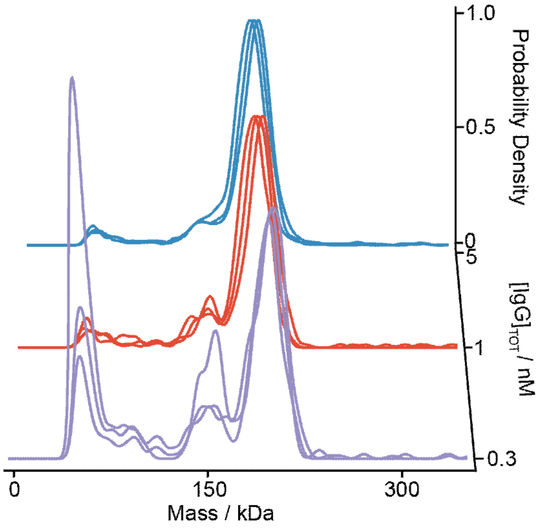

Supplementary Figure 14: Concentration screening of IgG-FcγIa complexes. IgG - FcγIa was mixed at a 1:1 ratio with final IgG concentration of 2.7 µM, followed by overnight incubation at room temperature. Samples were diluted (0.26, 1.3 and 4.4 nM) and measured after 9.6 - 18 min incubation time. With increasing dilution and increasing incubation time, we found a minimal increase in the unbound IgG peak intensity (apparent K_d = 80 ± 10 pM at 4.4 nM, 25 ± 6 pM at 1.5 nM and 18 ± 7 at 260 pM). From this, a K_d in the sub-nM range and an equilibration time >20 min can be estimated.

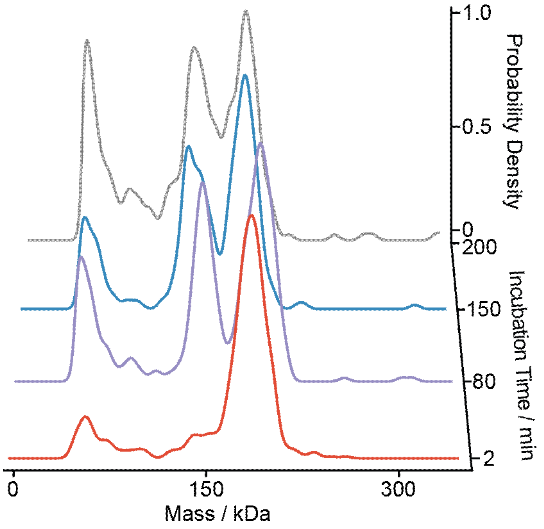

Supplementary Figure 15: Equilibration time screening for IgG-FcγIa. IgG -FcγIa were mixed at a 1:1 ratio with final IgG concentration of 2.7 μM, followed by overnight incubation at room temperature. Samples were diluted (0.28 and 0.21 nM) and measured after 2-200 min. Starting with an apparent K_d value of 200 fM after 2 min, K_d values are reaching a plateau-region after > 80 min incubation time, with K_d values between 60-100 pM.

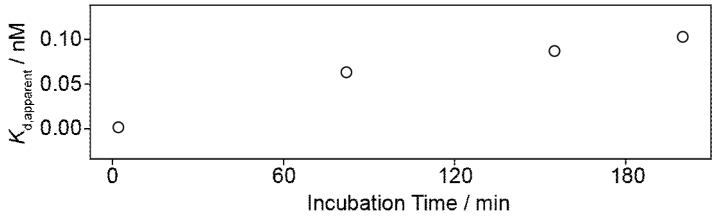

Supplementary Figure 16: *K*$_d$ values from equilibration time screening for IgG-FcγIa. Calculated from data in **Supplementary Figure 14**.

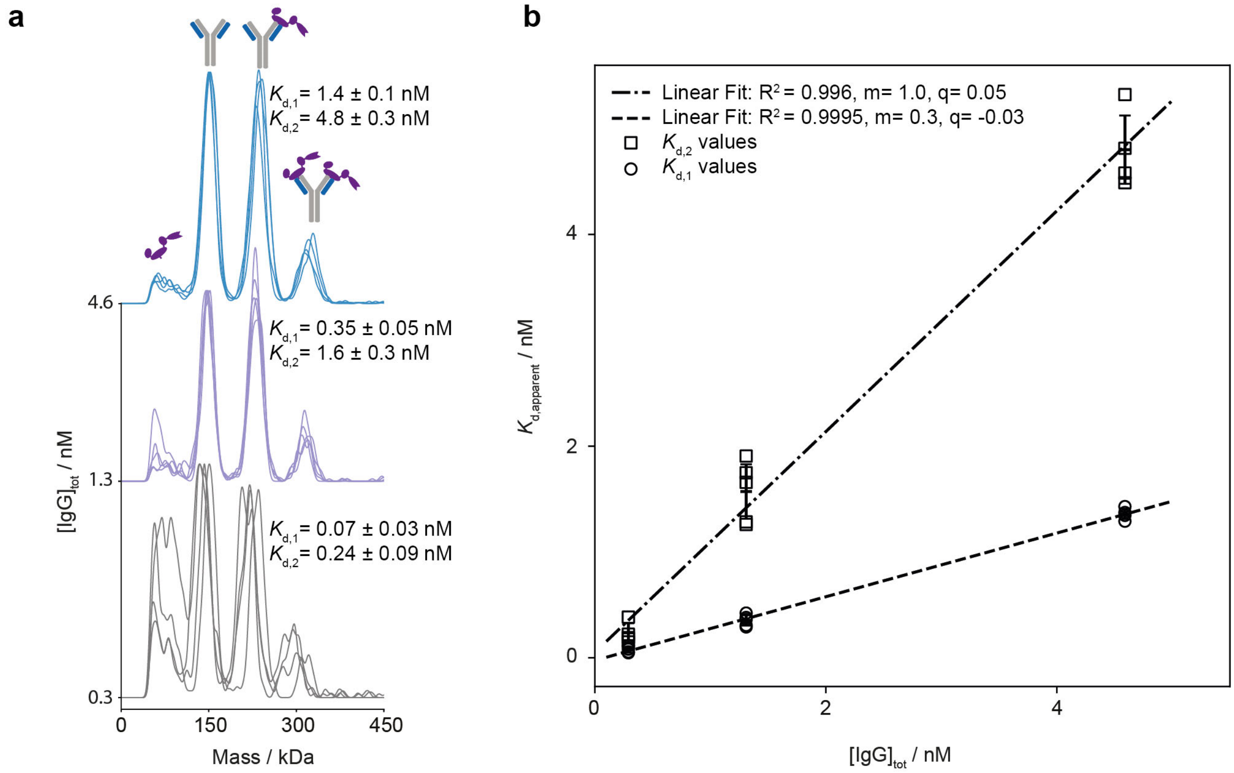

Supplementary Figure 17: Screening the trastuzumab-HER2 interaction. (**a**) 1.8 µM trastuzumab and 1.8 µM HER2 mixtures were equilibrated at room temperature overnight and diluted to desired pM-nM concentrations (0.3 nM, 1.3 nM and 4.6 nM). No significant differences in relative abundances of bound/unbound complexes were observed at various incubation times (2.5 – 100 min) for all concentrations. (**b**) Apparent K_d values showed a strong linear dependence on concentration, indicating non-equilibrium conditions and/or K_d values, which are exceeding our current dynamic concentration range/sensitivity of MP (i.e. sub-pM K_d). No valid K_d value could be determined but the data suggests very strong binding affinities of trastuzumab to HER2 (approximately < pM) and/or very slow off rates (> hrs). This example highlights the importance of concentration and equilibration time screening for accurate K_d measurements.

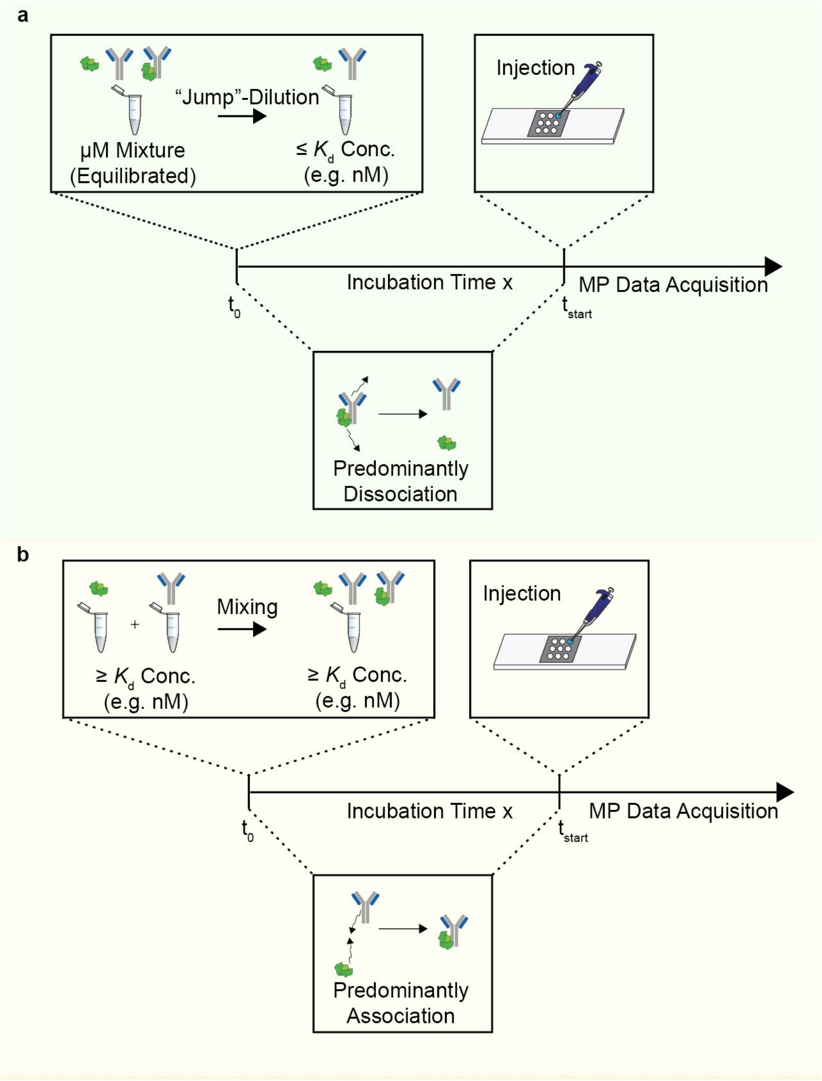

Supplementary Figure 18: Principle of dissociation and association measurements. A jump-dilution strategy for measuring K_d and kinetics. Dilution to sub-K_d concentrations results in dissociation of protein complexes over time (from t_0). After incubation time t_{start} we quantify the bound to unbound ratio with a MP measurement, typically lasting 30 s (for gaskets). Mixing at concentrations above the expected K_d, in association experiments permits the quantification of complexes that form from t_0. Importantly, non-specific protein adsorption is a factor in both methods, but more noticeable for association measurements.

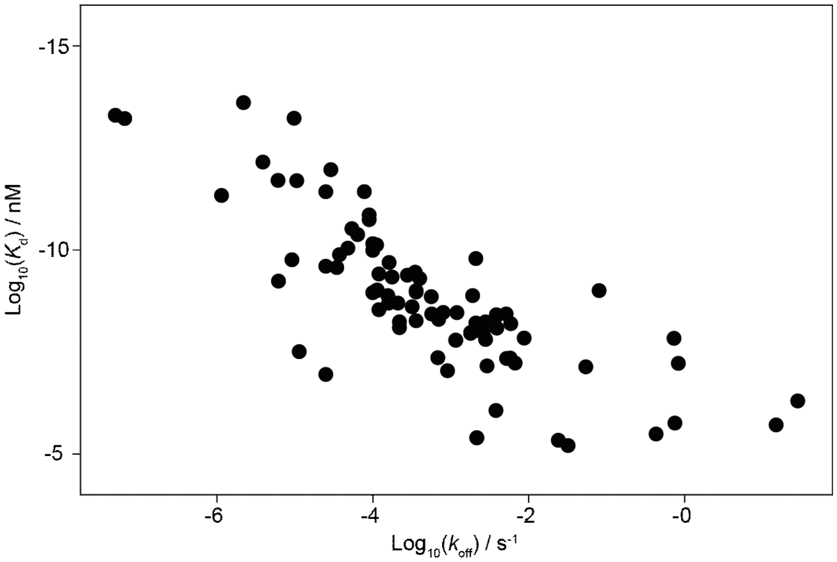

Supplementary Figure 19: Correlation of published K_d vs k_{off} values. Various biomolecular interactions measured by orthogonal techniques (e.g. SPR, BLI). Data is available in **Table 5**. These data show a general correlation between stronger binding affinities corresponding to slower k_{off} rates.

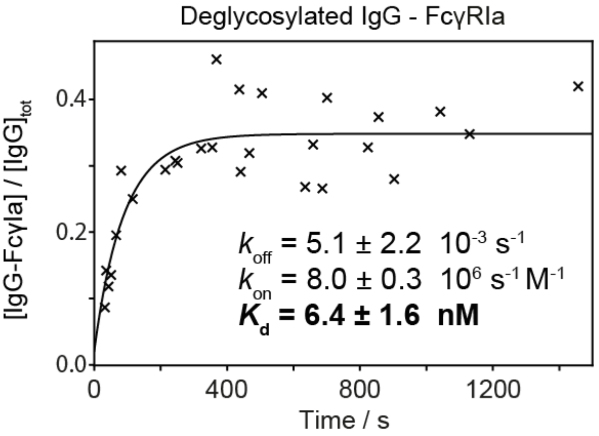

Supplementary Figure 20: Association measurements (k_{on}) of IgG$_{deglycosylated}$-FcγIa. IgG$_{deglycosylated}$-FcγIa was mixed at a 1:1 ratio with final concentrations of 4.9 nM for FcγIa and 4.6 nM for IgG$_{deglycosylated}$. Individual measurements (x) were taken after different incubation times (0.4 – 15 min). The k_{on}, k_{off} and K_d values were obtained from a non-linear fit (black line) to the experimental data and were in good agreement with values obtained from the dissociation experiment.

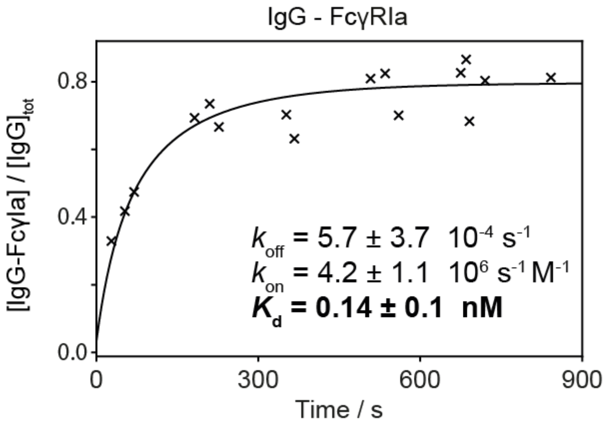

Supplementary Figure 21: Association measurements (k_{on}) of IgG-Fcγla. IgG-Fcγla were mixed at a 1:1 ratio with final concentrations of 3.0 nM for Fcγla and 3.1 nM for IgG. Individual measurements (x) were taken at different incubation times (0.5 – 44 min). The k_{on}, k_{off} and K_d values were obtained from a non-linear fit (black line) to the experimental data and were in good agreement with values obtained from the dissociation experiment.

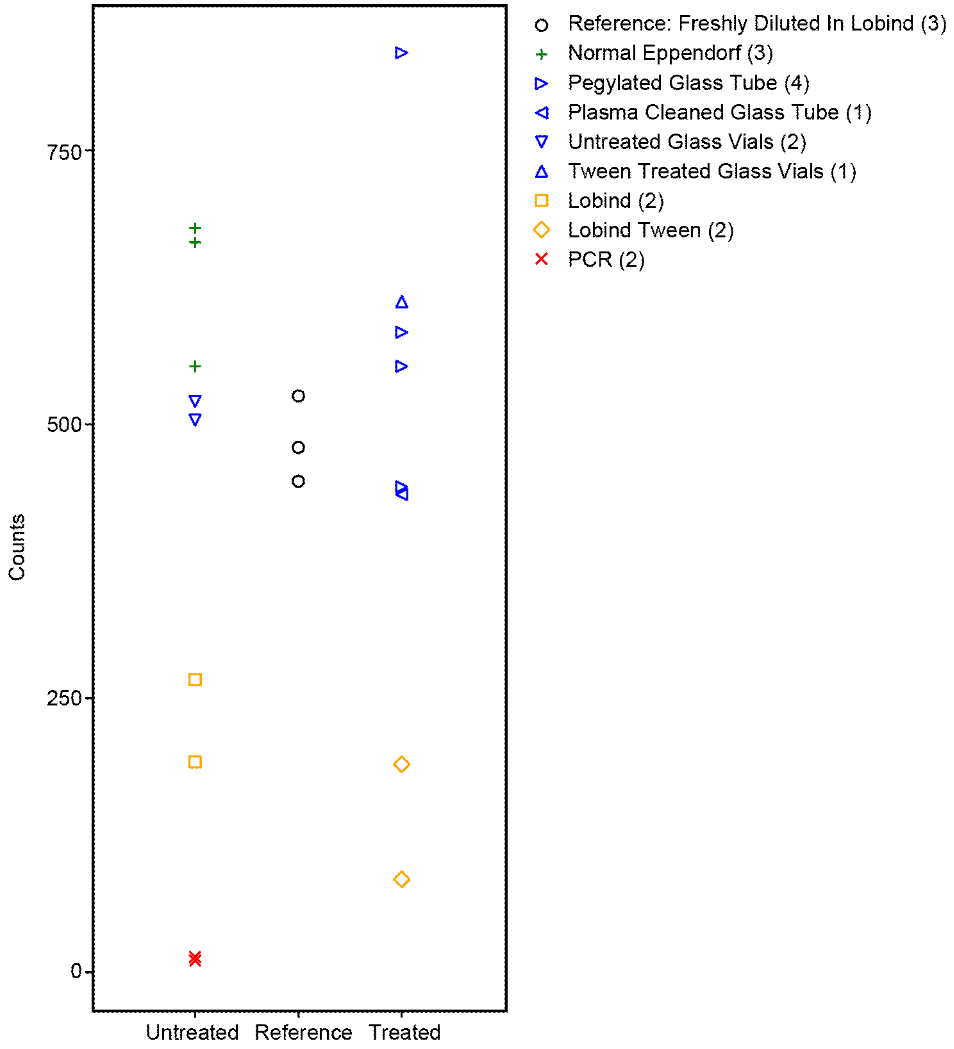

Supplementary Figure 22: Non-specific IgG adsorption to samples tubes. We freshly prepared 5 nM IgG in Eppendorf Lobind tubes to compare with the performance of other alternative materials, where IgG was stored for 20 hrs. Normal Eppendorf tubes were able to maintain the counts/concentration during this period. We saw good performance of glass vials, potentially due to their smaller surface-to-volume ratio. Eppendorf Lobind showed around 50% loss and PCR tubes >90% loss. We concluded from these results that normal Eppendorf tubes were most suitable for our experiments.

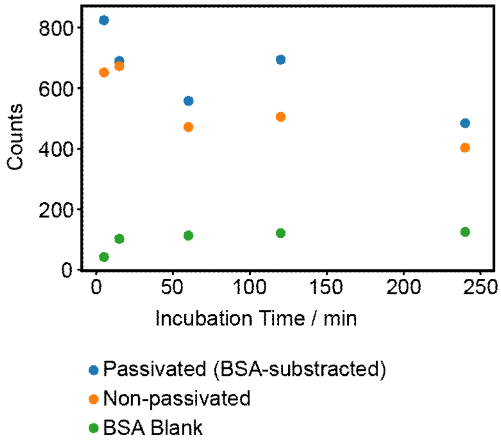

Supplementary Figure 23: Protein passivation of sample tubes with BSA. BSA showed no significant improvement to help maintaining concentration, i.e. counts, over time, potentially due to its high solubility.

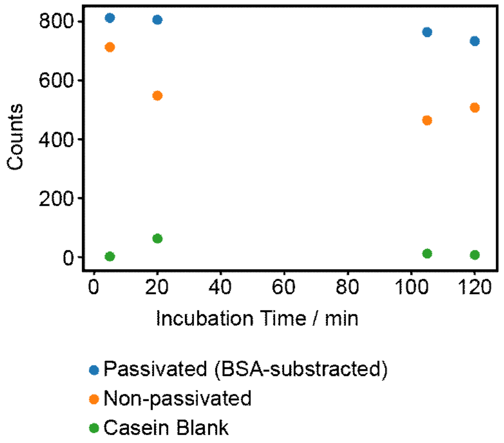

Supplementary Figure 24: Protein passivation of sample tubes with casein. Casein passivation helped maintain the concentration close to the initial level. Casein, seems to be ideal to passivate plastic surfaces, most likely because of its low solubility in water. Additionally, due to its low molecular weight (main species < 25 kDa), it does not cause interference in MP as it is below the detection limit of the current instrument.

a

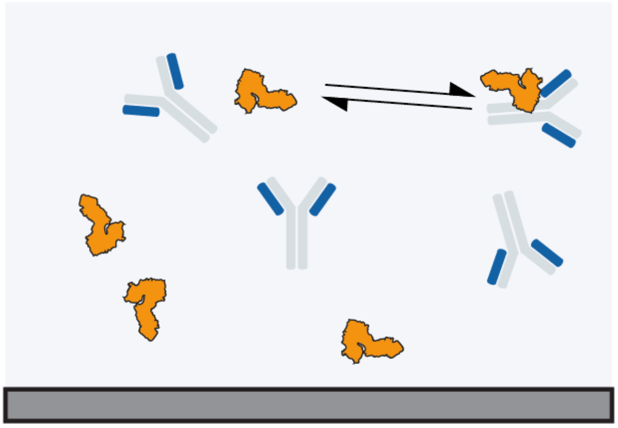

b

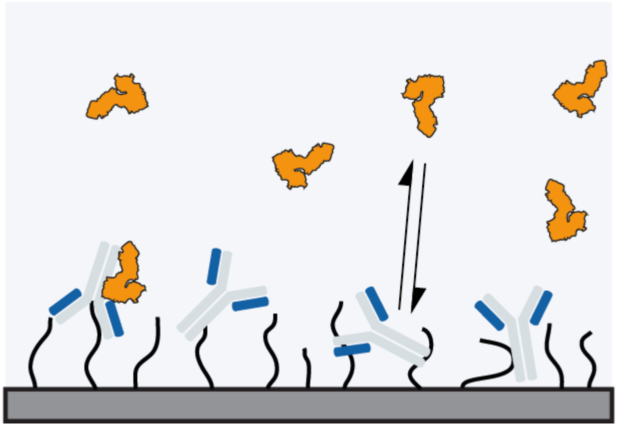

Supplementary Figure 25: Schematic of interactions in MP and SPR. (**a**) In-solution, label-free interactions occuring in MP. (**b**) Surface-immobilization (e.g. in a dextran matrix) and associated interactions in SPR. Differences in on-rates between SPR and MP (**Figure 2g & 2h** and **Supplementary Figure 20 & 21)** are attributed to mass transport, protein immobilization (i.e. orientation of IgG) and matrix effects [8]. Lower experimental on-rates, therefore, lead to greater calculated K_d values by SPR.

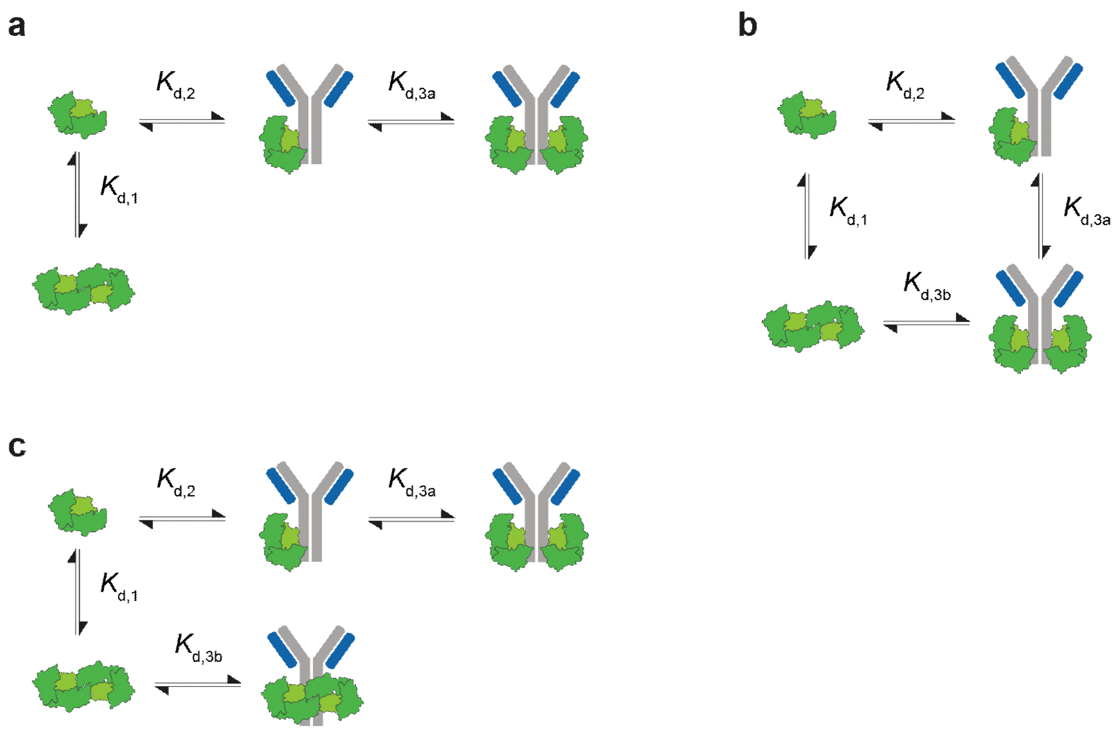

Supplementary Figure 26: Proposed binding models for IgG-FcRn interactions. Based on the existing literature we calculated binding affinities based on the free monomer binding model (a). The stoichiometries observed in our IgG-FcRn data would support also other models (**b, c**).

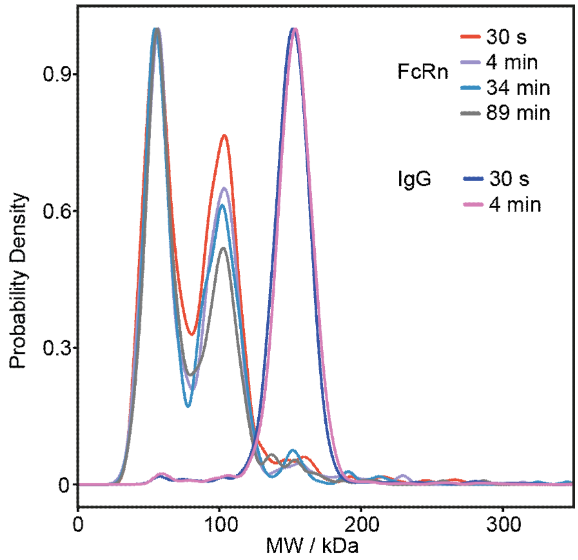

Supplementary Figure 27: Technical replicates of IgG and FcRn pH = 5. IgG (9 nM, purple) and FcRn (12 nM, red) measured at different time points. We can observe monomeric IgG at pH = 5 and FcRn present as monomer, dimer and in small quantities as trimer. The data suggests that FcRn reaches equilibrium within minutes (<30 min).

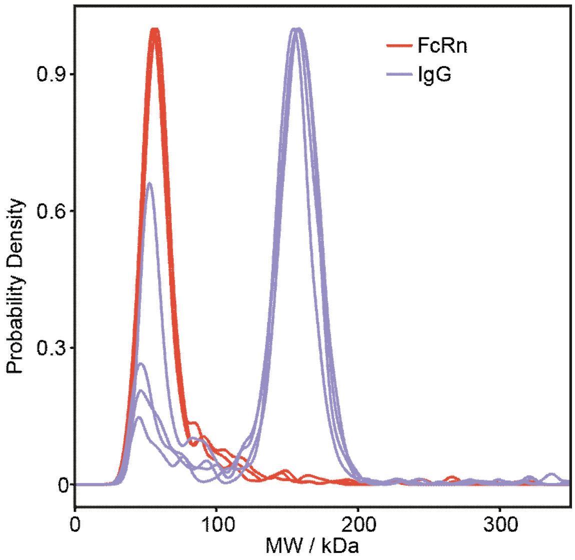

Supplementary Figure 28: Technical replicates of IgG and FcRn pH = 5.5. IgG (purple) was diluted from 3.6 µM to 2.5 nM and measured after different incubation times (0.4, 4.2, 10.9, 14.3 min). FcRn (red) was diluted from 7.1 µM to 4.5 nM and measured after 0.4, 4.2 and 15.7 min. Compared to pH = 5.0 we observe significantly less FcRn dimer.

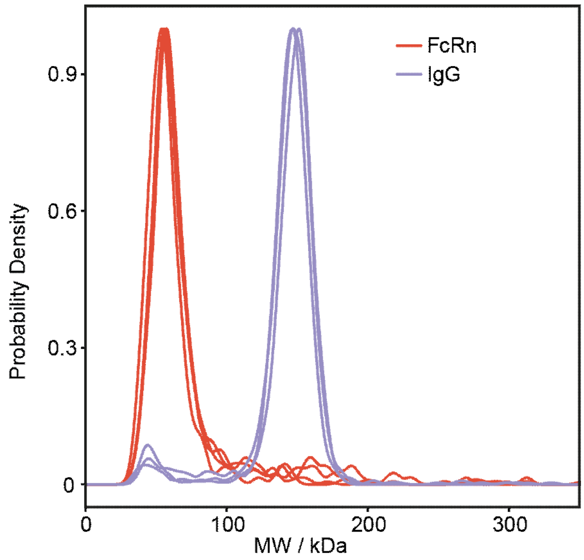

Supplementary Figure 29: Technical replicates of IgG and FcRn pH = 6.0. IgG (purple) was diluted from 2.0 µM to 4 nM and measured after different incubation times (0.7, 4.0, 7.0 min). FcRn (red) was diluted from 19.9 µM to 6.1 nM and measured after 0.4, 1, 3.8 and 12 min. Compared to pH = 5.0 we observe significantly less FcRn dimer.

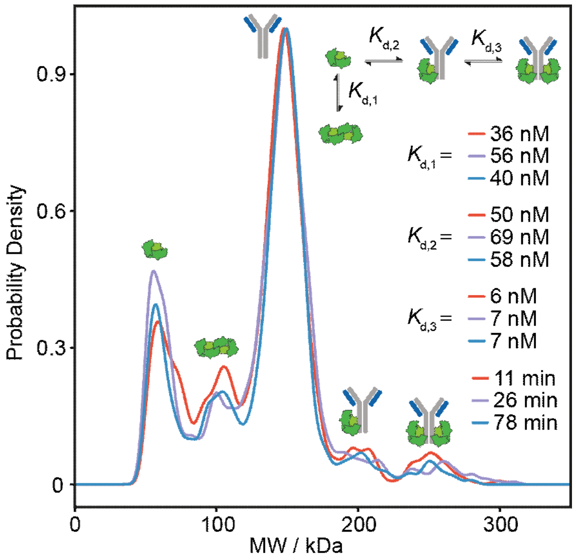

Supplementary Figure 30: Time point and K_d measurements of IgG-FcRn at pH = 5. IgG concentrations were 3 nM IgG and 6 nM FcRn. Measurements were taken after 11 (red), 26 (purple) and 78 (blue) minutes equilibration time. The IgG-FcRn at pH 5.0 showed predominantly unbound IgG (88 ± 1 %), minor amounts of IgG bound to one (7 ± 1%) and two (5 ± 1%) FcRns.

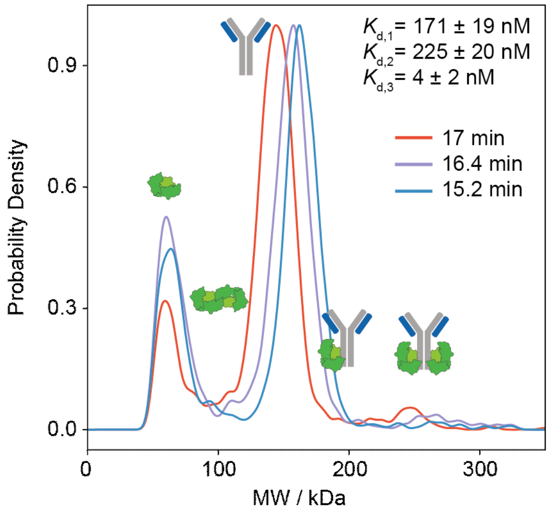

Supplementary Figure 31: Time point and K_d measurements of IgG-FcRn at pH = 5.5. IgG concentrations were 3 nM IgG and 6 nM FcRn. Measurements were taken at 17 (red), 16 (purple) and 15 (blue) minutes.

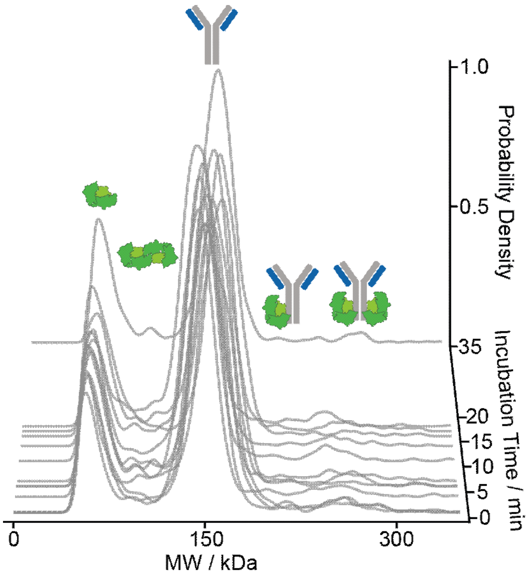

Supplementary Figure 32: Time course measurements of IgG-FcRn binding at pH = 5.5. Measurements were taken from 0.4 to 35 minutes at 3 nM IgG and 6 nM FcRn. Over the time course of 35 min no further dissociation of the bound species could be observed, suggesting that equilibrium is reached rapidly (<min).

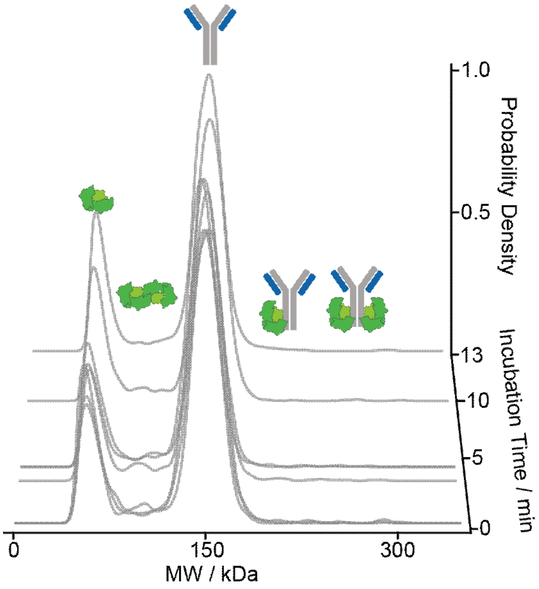

Supplementary Figure 33: Time course measurements of IgG-FcRn binding at pH = 6.0. Measurements were taken from 0.3 to 13 minutes at 3 nM IgG and 6 nM FcRn. Bound species were too low in abundance to be quantified. K_d values are expected to be above 200 nM.

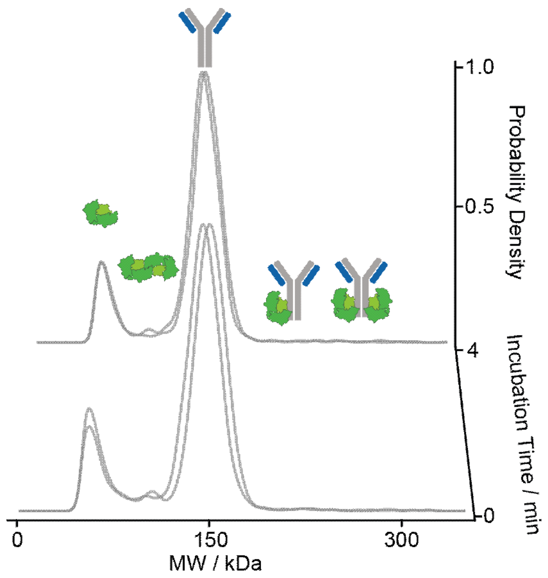

Supplementary Figure 34: Time course measurements of IgG-FcRn binding at pH = 7.0. Measurements were taken from 0.3 to 4 minutes at 3 nM IgG and 6 nM FcRn. Bound species were too low in abundance to be quantified. K_d values are expected to be above 200 nM.

11. Supplementary Table 5: Raw data of published biomolecular binding affinities.

#PDB	Affinity (M)	k_{off} (s^{-1})	Protein 1	Protein 2	Method	reference
2FTL_E_I	5.00E-14	5.00E-08	Bovine trypsin	BPTI	IASP	[9]
1TM1_E_I	7.00E-13	3.90E-06	Subtilisin BPN	Chymotrypsin inhibitor 2	IASP	
3W2D_A_HL	5.79E-10	6.18E-06	Staphylococcal enterotoxin B	3E2 fab	SPR	
1TM1_E_I	2.00E-12	1.06E-05	Subtilisin BPN	Chymotrypsin inhibitor 2	SFFL	
4HFK_A_BD	2.69E-10	3.46E-05	Tae4	Tai4	SPR	
5C6T_HL_A	1.30E-10	3.75E-05	1G2 fab	HCMV glycoprotein B	SPR	
4U6H_AB_E	9.00E-11	4.80E-05	M12B9 fab	Vaccinia L1	BI	
3HFM_HL_Y	3.00E-11	5.40E-05	HyHEL-10	HEW Lysozyme		
4CVW_A_C	4.20E-11	6.40E-05	Limit dextrinase	Limit dextrinase inhibitor	SPR	
2SIC_E_I	1.80E-11	9.00E-05	Subtilisin BPN	Streptomyces subtilisin inhibitor	IASP	
3HFM_HL_Y	7.00E-11	1.00E-04	HyHEL-10	HEW Lysozyme		
2VIR_AB_C	1.00E-09	1.10E-04	IgG1 lambda fab	Flu virus hemagglutinin	SPR	
3HFM_HL_Y	7.50E-11	1.12E-04	HyHEL-10	HEW Lysozyme		
1BJ1_HL_VW	2.90E-09	1.20E-04	Fab-12	VEGF		
1N8Z_AB_C	1.31E-09	1.56E-04	Herceptin	erbB-2	SPR	
4MNQ_ABC_DE	2.00E-09	1.60E-04	HLA-A2 plus telomerase peptide	ILA1 TCR	SPR	
3BT1_A_U	4.60E-10	1.77E-04	Urokinase-type plasminogen activator	Urokinase plasminogen activator surface receptor	SPR	
1GL0_E_I	2.00E-09	2.10E-04	Bovine alpha-chymotrypsin	PMP-D2v insect inhibitor		
4K71_A_BC	8.00E-09	2.20E-04	Human Serum Albumin	FcRn		
3NGB_HL_G	5.76E-09	2.20E-04	VRC01 fab	gp120	SPR	
2DSQ_I_G	4.14E-10	2.78E-04	IGF-I	IGFBP1	SPR	
4HSA_AB_C	2.46E-09	3.20E-04	Interleukin-17a	Interleukin-17 receptor A	SPR	
1N8Z_AB_C	3.50E-10	3.50E-04	Herceptin	erbB-2	SPR	
2B42_A_B	1.07E-09	3.60E-04	TAXI-I	B. subtilis endoxylanase	SPR	
1N8Z_AB_C	5.00E-10	4.00E-04	Herceptin	erbB-2	SPR	
1DAN_HL_UT	3.70E-09	5.70E-04	Factor VIIa	Tissue factor		
4JPK_HL_A	4.36E-08	6.83E-04	VRC01 fab	eOD-GT6	SPR	
2AJF_A_E	1.62E-08	1.16E-03	Human Angiotensin-converting enzyme 2	SARS spike protein receptor binding domain	SPR	
1CBW_FGH_I	1.10E-08	1.80E-03	Bovine alpha-chymotrypsin	BPTI	IASP	
1YY9_CD_A	1.31E-09	1.91E-03	Cetuximab fab	Epidermal growth factor receptor	SPR	
2I26_N_L	9.50E-09	2.00E-03	Type II IgNAR	HEW Lysozyme	SPR	
1IAR_A_B	1.62E-10	2.10E-03	Interleukin-4	Interleukin-4 receptor	SPR	
1DAN_HL_UT	6.16E-09	2.10E-03	Factor VIIa	Tissue factor		
2VIS_AB_C	4.00E-06	2.16E-03	IgG1 lambda fab	Flu virus hemagglutinin		
1MHP_HL_A	1.07E-08	2.60E-03	AQC2 fab	Integrin alpha-1	SPR	

1WQJ_I_B	5.78E-09	2.79E-03	IGF-I	IGF-1R	SPR	
3BP8_A_C	4.14E-09	3.85E-03	Mlc transcription regulator	PTS glucose-specific enzyme EIICB	SPR	
1NMB_N_LH	4.55E-08	5.20E-03	Subtype N9 neuraminidase	Antibody NC10	SPR	
5F4E_A_B	5.90E-08	6.70E-03	Sperm-egg fusion protein Izumo	Sperm-egg fusion protein Juno	BI	
1JRH_LH_I	1.44E-08	8.75E-03	mAbs A6	Interferon gamma receptor	SPR	
4K71_A_BC	6.20E-06	3.20E-02	Human Serum Albumin	FcRn		
2ptc	6.00E-14	6.60E-08	Trypsinogen	BPTI	spectrophotometric assays	
1jiw	4.60E-12	1.15E-06	Alkaline metallo-proteinase	Proteinase inhibitor	spectrophotometric assays	
1emv	2.44E-14	2.20E-06	Colicin E9 nuclease	Im9 immunity protein	stopped-flow fluorescence	
2sni	1.97E-12	6.10E-06	Subtilisin	Chymotrypsin inhibitor 2	spectrophotometric assays	
1t6b	1.74E-10	9.20E-06	Anthrax protective antigen	Anthrax toxin receptor	SPR and FRET	
1dfj	5.90E-14	9.80E-06	Ribonuclease A	Rnase inhibitor	RNAse assay	
1ffw	3.10E-08	1.14E-05	Chemotaxis protein CheY	Chemotaxis protein CheA	SPR	
1fsk	2.50E-10	2.50E-05	Fab - Birch pollen antigen Bet V1	Birch pollen antigen Bet V1		
1jmo	1.12E-07	2.50E-05	Heparin cofactor	Thrombin	spectrophotometric assays	
1ppe	3.73E-12	2.50E-05	Trypsinogen	CMTI-1 squash inhibitor	spectrophotometric assays	
1mah	1.07E-12	2.90E-05	Acetylcholinesterase	Fasciculin	spectrophotometric assays	
1eer	3.71E-12	7.80E-05	Erythropoietin	EPO receptor	SPR	
2sic	1.38E-11	9.00E-05	Subtilisin	Streptomyces subtilisin inhibitor	spectrophotometric assays	
1jps	1.02E-10	0.0001	Fab D3H44	Tissue factor	spectrophotometric assays	
2i25	1.11E-09	0.0001	Shark single domain antigen receptor	HEW lysozyme	SPR	[10]
2vir	1.00E-09	0.00011	Fab	Flu virus hemagglutinin	SPR	
2i9b	9.58E-10	0.000114	uPAR surface receptor	Urokinase-type plasminogen activator	SPR	
1jtg	3.87E-10	0.00012	β-lactamase inhibitor protein	β-lactamase TEM-1	spectrophotometric assays	
1gl1	2.03E-10	0.000162	Chymotrypsin	PMP-C (LCMI II)	spectrophotometric assays	
1jwh	5.41E-09	0.00036	Casein kinase II β chain	Casein kinase II α chain	SPR	
2b42	9.97E-10	0.00036	Xylanase	Xylanase inhibitor	SPR	
2gox	1.39E-09	0.000563	Complement C3d fragment	Staphylococcus aureus Efb-C	SPR	
1gxd	5.00E-09	0.0007	ProMMP2 type IV collagenase	Metalloproteinase inhibitor 2	SPR	
1kxq	3.39E-09	0.0008	Camel VHH - Pancreatic α-amylase	Pancreatic α-amylase	IAsys	
1mlc	9.10E-08	0.00091	Fab44.1	HEW lysozyme	SPR	
2ajf	1.63E-08	0.00116	Angiotensin-converting enzyme 2	SARS spike protein receptor binding domain	SPR	
1e6j	3.43E-09	0.0012	Fab 13B5	HIV-1 capsid protein p24	SPR	
1cbw	1.06E-08	0.0018	Chymotrypsin	BPTI	spectrophotometric assays	
2vis	4.00E-06	0.00216	Fab	1GIG LH	SPR	

1kac	1.56E-08	0.0028	Adenovirus fiber knob protein	Adenovirus receptor	SPR	
1p2c	6.94E-08	0.00292	FabF10.6.6	HEW lysozyme	IAsys	
1e6e	8.57E-07	0.0038	Adrenoxin reductase	Adrenoxin	SPR	
3bp8	3.87E-09	0.00385	Mlc transcription regulator	PTS glucose-specific enzyme EIICB	SPR	
2b4j	8.21E-09	0.0039	Integrase (HIV-1)	PC4 and SFRS1 interacting protein	fluorescence assay	
1vfb	3.70E-09	0.00514	Fv D1.3	HEW lysozyme	stopped-flow fluorescence	
1kkl	4.46E-08	0.0058	HPr kinase C-ter domain	HPr	SPR	
1xu1	6.39E-09	0.00589	TNF domain of APRIL	TACI CRD2 domain	SPR	
1klu	4.60E-06	0.02399999	MHC class 2 HLA-DR1	Staphylococcus enterotoxin C3	SPR	
1ktz	7.30E-08	0.05400003	TGF-β	TGF-β receptor	SPR	
2oza	9.88E-10	0.08	MAP kinase 14	MAP kinase-activated protein kinase 2	stopped-flow fluorescence	
1mq8	3.23E-06	0.42999995	ICAM-1 domain 1-2	Integrin α-L I domain	SPR	
2wpt	1.46E-08	0.73000024	Colicin E9 nuclease	Im2 immunity protein	stopped-flow fluorescence	
1e4k	1.74E-06	0.74999995	FC fragment of human IgG 1	Human FCGR III	SPR	
	6.00E-08	0.83	Colicin E9 nuclease	Im2 immunity protein	stopped-flow fluorescence	
1lfd	1.94E-06	14.8999908	Ras.GNP	RalGDS Ras-interacting domain	stopped-flow fluorescence	
	5.00E-07	28.2	Colicin E9 nuclease	Im8 immunity protein	stopped-flow fluorescence	

Index: EI (enzyme & inhibitor), ES (enzyme & substrate), ER (enzyme & receptor), OR (other & receptor), OX (other & miscellaneous), AB (antibody & antigen), SPR (surface plasmon resonance), IAsys (resonant mirror biosensor) , FRET (Förster resonance energy transfer microscopy), BI (biolayer interferometry), SFFL (stopped flow fluorescence).

12. Equations

Calculation of K_d values from relative abundances measured with MP.
For the interactions of IgG - Fcγla and deglycosylated IgG- Fcγla we obtain the following equations:
Mass balance:

(1) $$[IgG]_{total} = [IgG]_{unbound} + [IgG]_{bound}$$

$[IgG]_{total}$, $[IgG]_{unbound}$ and $[IgG]_{bound}$ are IgG molar concentrations

Calculation of conversion factor:

(2) $$f_{conversion} = \frac{[IgG]_{total}}{counts(IgG_{unbound})+counts(IgG_{bound})}$$

where $counts(IgG_{unbound})$ and $counts(IgG_{bound})$ are the counts obtained from Gaussian fits to the mass histograms, $f_{converesion}$ is the conversion factor which converts counts into molar concentrations

Conversion of counts to molar concentrations:

(3) $$[\mathrm{IgG}]_{\mathrm{bound}} = \mathrm{counts}\ (\mathrm{IgG}_{\mathrm{bound}}) * f_{conversion}$$

(4) $$[\mathrm{IgG}]_{\mathrm{unbound}} = \mathrm{counts}\ (\mathrm{IgG}_{\mathrm{unbound}}) * f_{conversion}$$

(5) $$[\mathrm{Fc\gamma Ia}]_{\mathrm{unbound}} = [\mathrm{Fc\gamma Ia}]_{\mathrm{total}} - [\mathrm{IgG}]_{\mathrm{bound}}$$

Due to variable noise contributions in the low molecular weight range (<80 kDa), we have to calculate the molar concentration of Fcγla via **equation 5**. The K_d is then obtained from **equation 6**.

(6) $$K_{\mathrm{d}} = \frac{[\mathrm{Fc\gamma Ia}]_{\mathrm{unbound}} * [\mathrm{IgG}]_{\mathrm{unbound}}}{[\mathrm{IgG}]_{\mathrm{bound}}}$$

For the interactions of IgG-FcRn we obtain the following equation:

Mass balance:

(7) $$[IgG]_{total} = [IgG]_{unbound} + [IgG]_{single\ bound} + [IgG]_{double\ bound}$$

Calculation of conversion factor:

(8) $$f_{converesion} = \frac{[IgG]_{total}}{counts(IgG_{unbound})+counts(IgG_{single\ bound})+counts(IgG_{double\ bound})}$$

Conversion of counts to molar concentrations:

(9) $$[\mathrm{IgG}]_{\mathrm{bound}} = \mathrm{counts}\ (\mathrm{IgG}_{\mathrm{bound}}) * f_{conversion}$$

(10) $$[\mathrm{IgG}]_{\mathrm{single\ bound}} = \mathrm{counts}\ (\mathrm{IgG}_{\mathrm{single\ bound}}) * f_{conversion}$$

(11) $$[\mathrm{IgG}]_{\mathrm{double\ bound}} = \mathrm{counts}\ (\mathrm{IgG}_{\mathrm{double\ bound}}) * f_{conversion}$$

(12) $$[FcRn_{dimer}] = \mathrm{counts}\ (\mathrm{FcRn}_{\mathrm{dimer}}) * f_{conversion}$$

(13) $[FcRn_{monomer}] = [FcRn]_{total} - 2 * [FcRn_{dimer}] - [\mathrm{IgG}]_{\text{single bound}} - 2 * [\mathrm{IgG}]_{\text{double bound}}$

Calculation of K_d values for interactions:

(14) $K_1 = \frac{[FcRN_{monomer}]^2}{[FcRN_{dimer}]}$

(15) $K_2 = \frac{[FcRN_{monomer}]*[IgG]_{unbound}}{[\mathrm{IgG}]_{\text{single bound}}}$

(16) $K_{3a} = \frac{[FcRN_{monomer}]*[\mathrm{IgG}]_{\text{single bound}}}{[\mathrm{IgG}]_{\text{double bound}}}$

(17) $K_{3b} = \frac{[FcRN_{dimer}]*[IgG]_{unbound}}{[\mathrm{IgG}]_{\text{double bound}}}$

(18) $K_{3,app} = K_{3a} * K_{3b}$

Kinetic experiments of IgG - FcγIa and deglycosylated IgG- FcγIa:

For association:

At t=0:

(19) $[\mathrm{Fc\gamma Ia}] = [\mathrm{Fc\gamma Ia}]_0 = [\mathrm{Fc\gamma Ia}]_{tot}$

(20) $[IgG_{unbound}] = [IgG_{unbound}]_0 = [IgG]_{tot}$

(21) $[IgG_{bound}] = [IgG_{bound}]_0 = 0$

At t>0:

(22) $[IgG_{unbound}] = [IgG_{unbound}]_0 - x_t$

(23) $[\mathrm{Fc\gamma Ia}] = [\mathrm{Fc\gamma Ia}]_0 - x_t$

(24) $[IgG_{bound}] = [IgG_{bound}]_0 + x_t$

(25) $K_d = \frac{k_{off}}{k_{on}}$

(26) $\frac{d[IgG_{bound}]}{dt} = k_{on} * [\mathrm{Fc\gamma Ia}] * [IgG_{unbound}] - k_{off} * [IgG_{bound}]$

(27) $\frac{d[IgG_{unbound}]}{dt} = \frac{d[\mathrm{Fc\gamma Ia}]}{dt} = -\frac{d[IgG_{bound}]}{dt}$

(28) $\frac{dx}{dt} = k_{on}([\mathrm{Fc\gamma Ia}]_0 - x_t) * ([IgG_{unbound}]_0 - x_t) - k_{off} * ([IgG_{bound}]_0 + x_t)$

(29) $$\frac{counts(IgG_{bound})}{counts(IgG_{tot})} = \frac{x_t}{[IgG]_{tot}}$$

For dissociation:
Calculation of the concentrations after equilibration of stock mixtures (ca. µM concentrations):

(30) $$[IgG_{unbound}]_{eq,conc} = [\mathrm{Fc\gamma Ia}]_{eq,conc}$$

(31) $$[IgG_{unbound}]_{eq,conc} = [IgG]_{tot,conc} - [IgG_{bound}]_{eq,conc}$$

(32) $$K_d = \frac{([IgG]_{tot,conc} - [IgG_{bound}]_{eq,conc})^2}{[IgG_{bound}]_{eq,conc}}$$

Using the known dilution factor (from dilutiong the stock mixtures (ca. µM) to nM/pM concentrations):

At t=0:

(33) $$[\mathrm{Fc\gamma Ia}] = [\mathrm{Fc\gamma Ia}]_0 = \frac{[\mathrm{Fc\gamma Ia}]_{eq,conc}}{f_{dilution}}$$

(34) $$[IgG_{unbound}] = [IgG_{unbound}]_0 = \frac{[IgG_{unbound}]_{eq,conc}}{f_{dilution}}$$

(35) $$[IgG_{bound}] = [IgG_{bound}]_0 = \frac{[IgG_{bound}]_{eq,conc}}{f_{dilution}}$$

At t>0:

(36) $$[IgG_{unbound}] = [IgG_{unbound}]_0 + x_t$$

(37) $$[\mathrm{Fc\gamma Ia}] = [\mathrm{Fc\gamma Ia}]_0 + x_t$$

(38) $$[IgG_{bound}] = [IgG_{bound}]_0 - x_t$$

$$K_D = \frac{k_{off}}{k_{on}}$$

Differential equation describing the dissociation:

(39) $$\frac{d[IgG_{bound}]}{dt} = -k_{on} * [\mathrm{Fc\gamma Ia}] * [IgG_{unbound}] + k_{off} * [IgG_{bound}]$$

(40) $$\frac{d[IgG_{unbound}]}{dt} = \frac{d[\mathrm{Fc\gamma Ia}]}{dt} = -\frac{d[IgG_{bound}]}{dt}$$

(41) $$\frac{dx}{dt} = k_{on}([\mathrm{Fc\gamma Ia}]_0 + x_t) * ([IgG_{unbound}]_0 + x_t) - k_{off} * ([IgG_{bound}]_0 - x_t)$$

(42) $$\frac{counts(IgG_{bound})}{counts(IgG_{tot})} = \frac{[IgG_{bound}]_0 - x_t}{[IgG]_{tot}}$$

References

[1] Y. Watanabe, S. Vasiljevic, J. D. Allen, G. E. Seabright, H. M. E. Duyvesteyn, K. J. Doores, M. Crispin, W. B. Struwe, *Anal. Chem.* **2018**, *90*, 7325–7331.
[2] Y. Wu, A. P. West, H. J. Kim, M. E. Thornton, A. B. Ward, P. J. Bjorkman, *Cell Rep.* **2013**, *5*, 1443–1455.
[3] G. Young, N. Hundt, D. Cole, A. Fineberg, J. Andrecka, A. Tyler, A. Olerinyova, A. Ansari, E. G. Marklund, M. P. Collier, et al., *Science.* **2018**, *360*, 423–427.
[4] D. Cole, G. Young, A. Weigel, A. Sebesta, P. Kukura, *ACS Photonics* **2017**, *4*, 211–216.
[5] M. P. Nicholas, L. Rao, A. Gennerich, *Methods Mol. Biol.* **2014**, *1136*, 137–169.
[6] M. T. Marty, A. J. Baldwin, E. G. Marklund, G. K. A. Hochberg, J. L. P. Benesch, C. V. Robinson, *Anal. Chem.* **2015**, *87*, 4370–4376.
[7] M. Kiyoshi, J. M. M. Caaveiro, T. Kawai, S. Tashiro, T. Ide, Y. Asaoka, K. Hatayama, K. Tsumoto, *Nat. Commun.* **2015**, *6*, 4–6.
[8] P. Schuck, H. Zhao, *Methods Mol. Biol.* **2010**, *627*, 15–54.
[9] J. Jankauskaitė, B. Jiménez-García, J. Dapkūnas, J. Fernández-Recio, I. Moal, *Bioinformatics* **2018**, *35*, 462–469.
[10] I. H. Moal, P. A. Bates, *PLoS Comput. Biol.* **2012**, *8*, e1002351.

Author Contributions

Conceptualization: F.S., W.S., and P.K.; Investigation: F.S., E.F. and V.P.; Formal Analysis: F.S. and M.G.; Writing – Original Draft: F.S., W.S., and PK; Writing – Review & Editing: all authors; Visualization: F.S., W.S. and J.L.P.B.; Supervision: W.S. and P.K.
